# Deciphering the signaling mechanisms of the plant cell wall degradation machinery in *Aspergillus oryzae*

**DOI:** 10.1186/s12918-015-0224-5

**Published:** 2015-11-14

**Authors:** D.B.R.K. Gupta Udatha, Evangelos Topakas, Margarita Salazar, Lisbeth Olsson, Mikael R. Andersen, Gianni Panagiotou

**Affiliations:** The Norwegian Structural Biology Centre, Department of Chemistry, Faculty of Science and Technology, University of Tromsø, N-9037 Tromsø, Norway; Section for Genetics and Evolutionary Biology, Department of Biosciences, University of Oslo, Oslo, Norway; Biotechnology Laboratory, School of Chemical Engineering, National Technical University of Athens, 5 Iroon Polytechniou Str., Zografou Campus, Athens, 15780 Greece; Department of Chemical and Biological Engineering, Chalmers University of Technology, Gothenburg, Sweden; Wallenberg Wood Science Center, Chalmers University of Technology, SE-412 96 Gothenburg, Sweden; Department of Systems Biology, Technical University of Denmark, Søltofts plads 223, DK-2800 Lyngby, Denmark; School of Biological Sciences, The University of Hong Kong, Kadoorie Biological Sciences Building, Hong Kong, China

**Keywords:** Gene expression, Transcriptional activators, Lignocellulotytic enzymes, Molecular modeling, Inducers, Metabolic network

## Abstract

**Background:**

The gene expression and secretion of fungal lignocellulolytic enzymes are tightly controlled at the transcription level using independent mechanisms to respond to distinct inducers from plant biomass. An advanced systems-level understanding of transcriptional regulatory networks is required to rationally engineer filamentous fungi for more efficient bioconversion of different types of biomass.

**Results:**

In this study we focused on ten chemically defined inducers to drive expression of cellulases, hemicellulases and accessory enzymes in the model filamentous fungus *Aspergillus oryzae* and shed light on the complex network of transcriptional activators required. The chemical diversity analysis of the inducers, based on 186 chemical descriptors calculated from the structure, resulted into three clusters, however, the global, metabolic and extracellular protein transcription of the *A. oryzae* genome were only partially explained by the chemical similarity of the enzyme inducers. Genes encoding enzymes that have attracted considerable interest such as cellobiose dehydrogenases and copper-dependent polysaccharide mono-oxygenases presented a substrate-specific induction. Several homology-model structures were derived using *ab-initio multiple threading alignment* in our effort to elucidate the interplay of transcription factors involved in regulating plant-deconstructing enzymes and metabolites*.* Systematic investigation of metabolite-protein interactions, using the 814 unique reactants involved in 2360 reactions in the genome scale metabolic network of *A. oryzae,* was performed through a two-step molecular docking against the binding pockets of the transcription factors AoXlnR and AoAmyR. A total of six metabolites viz., sulfite (H_2_SO_3_), sulfate (SLF), uroporphyrinogen III (UPGIII), ethanolamine phosphate (PETHM), D-glyceraldehyde 3-phosphate (T3P1) and taurine (TAUR) were found as strong binders, whereas the genes involved in the metabolic reactions that these metabolites appear were found to be significantly differentially expressed when comparing the inducers with glucose.

**Conclusions:**

Based on our observations, we believe that specific binding of sulfite to the regulator of the cellulase gene expression, AoXlnR, may be the molecular basis for the connection of sulfur metabolism and cellulase gene expression in filamentous fungi. Further characterization and manipulation of the regulatory network components identified in this study, will enable rational engineering of industrial strains for improved production of the sophisticated set of enzymes necessary to break-down chemically divergent plant biomass.

**Electronic supplementary material:**

The online version of this article (doi:10.1186/s12918-015-0224-5) contains supplementary material, which is available to authorized users.

## Background

The development of alternatives to fossil fuels as an energy source is an urgent global priority, whereas a significant amount of attention is to replace petroleum with plant biomass as energy source [1]. According to estimations, biomass synthesis, as a result of biosynthesis increases approximately on 1.5 trillion tons annually, offering the possibility of a potential feedstock for biofuels and new biomaterial portfolios [2]. Lignocellulose in vascular plant cell walls is composed of cellulose, hemicellulose and lignin with the relative proportions of the three to be dependent on the material source [3]. The three major polymers in the plant cell wall build a complex network structure, which largely contributes to its recalcitrant nature. Several technologies, from standard combustion to complex bioconversion processes, have been applied to access the energy stored in the plant cell wall polymers and the clear objective now is to make this process cost competitive in today’s markets [4]. However, the plant cell wall and its metabolism are currently poorly understood due to its complex structure and biological recalcitrance, which is also largely responsible for the high cost of lignocelluloses conversion [5].

It is remarkable that numerous microorganisms in nature, mostly bacteria and fungi, are capable of producing biomass-degrading enzymes that are evolved as individual degraders or as part of microbial communities in some ecosystems [6]. Since these microorganisms are a treasure trove of enzymatic tools, increasing our knowledge of the biochemical machinery used by them for the breakdown of biomass will open new avenues for the development of biologically based processes [7]. Faced with the complex regulation of the production for a large number of enzymes and the fact that many enzymes with diverse functionality are needed for the utilization of certain polysaccharides, it is a necessity to apply a systems-wide approach for mapping the regulatory network [8]. Being able to examine the entire enzymatic toolbox at once, using gene expression signatures, can shed light on regulatory mechanisms that might not be possible to bridge with a hypothesis driven approach.

Filamentous fungi belonging to the genus *Aspergillus* have been used in the production of food ingredients, pharmaceuticals and enzymes, while the recent achievements made in *Aspergillus* biotechnology potentiate a dominant place among microbial cell factories [9]. Especially with regard to *Aspergillus oryzae,* the interest has increased due to its prominent potential for the secretory production of various enzymes, such as industrial enzymes (α-amylases, proteases, lipases) with use in modern biotechnology [10]. The sequencing of the *A. oryzae* genome showed that is larger than those of *A. fumigatus* and *A. nidulans* by approximately 34 and 29 %, respectively, but comparable to its close relatives, *A. flavus* and *A. niger.* The genome sequence of *A. oryzae* revealed 12,074 annotated genes, however, the number of hypothetical proteins accounted for more than 50 % of the annotated genes [11]. The genome sequence of *A. oryzae* has also revealed striking metabolic diversity, which obviously indicates the potential of the organism for further biotechnological applications as a source of many industrial enzymes other than amylases and proteases [12]. In addition the finding that a large number of pectinolytic genes exist in the *A. oryzae* genome suggested that *A. oryzae* may be a domesticated version of wild plant pathogens such as *A. flavus*, and thus might have a higher, than initially expected, number of plant cell wall degrading enzymes. However, despite the considerable commercial importance of this fungus, knowledge of *A. oryzae* biology has been very limited mainly due to difficulties in studying the organism by conventional genetic methods.

**Fig. 1:**
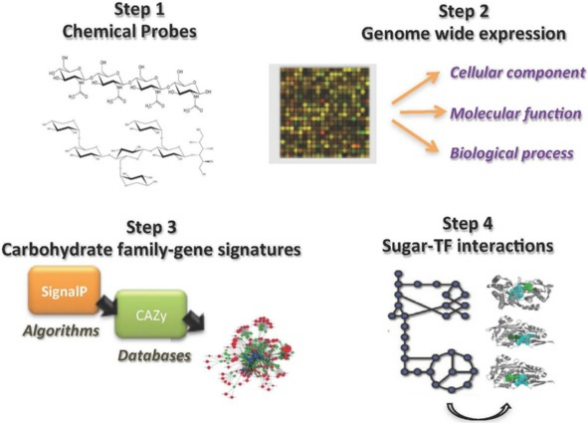
Graphical abstract of the work. (*Step-1*) selection of oligosaccharides with different chemical structures; (*Step-2*) monitor the genome-wide expression profile induced by the chemical probes; (*Step-3*) identification of the plant biomass degrading capacity; (*Step-4*) studying the interactome profile of the transcriptional regulators of carbohydrate active enzymes

In this study the interplay of plant cell wall components with *A. oryzae*'s metabolic, secretome and signaling pathways were investigated from a chemical perspective using an integrated analysis of the transcriptome profile. We were able to identify known and novel hydrolases, genes and pathways that might be involved in triggering or contributing to high protein production but also observe the induction of hydrolytic enzymes that cannot be justified from the chemical features of the substrates. We also showed the novel regulatory loops of the transcription factors controlling hydrolase production in *A. oryzae* using docking and protein-protein interaction networks (Fig. 1).

## Results and discussion

### Summary of transcription analysis datasets

To obtain a global view of the *A. oryzae* transcriptome activated for plant biomass conversion, mRNA from mycelium after a 2 h-induction on 10 different carbohydrate active enzyme inducers (di- and oligo- saccharides) was subjected to custom-designed NimbleGen microarray analysis. Cellohexaose (O-CHE), mannohexaose (O-MHE), xylopentaose (O-XPE), arabinoheptaose (O-AHP), *1,3:1,4*-β-glucohexaose (O-BGHEXA), *6*^*3*^-α-D-glucosyl-maltotriosyl-maltotriose (O-GMH), *6*^*1*^-α-D-galactosyl-mannotriose (O-GM3), xyloglucan (*X*_*3*_*Glc*_*4*_-borohydride reduced; O-X3G4R), turanose (TYR) and sophorose (SOP) were used to induce the plant polysaccharide degradation machinery of *A. oryzae.* Monosaccharides were not selected in our study since they have been extensively investigated especially for various Aspergilli species, including *A. oryzae*. Medium- to large-sized xylo-oligosaccharides were used in the study by Miyazaki et al, as a xylan-derived induction signal for xylanase activity [13]. Xylopentaose was used in the report by Gilad et al, where integration of transcriptome and proteome data was applied to develop a mechanistic model of the substrate utilization [14]. Even though for cellohexaose, that was selected in our study, there are no reports for acting as a possible enzyme inducer, other cello-oligosaccharides (cellotriose, cellotetraose and cellopentaose) were investigated as inducers of the genes encoding cellobiohydrolases by Suzuki et al [15]. For the other oligosaccharides used in our study their role as enzyme inducers has not been investigated, however, since most of the carbohydrate acting enzymes are exo-acting, we hypothesized that they are mainly aiming at oligosaccharides (especially as no evidence for oligosaccharide transport has been reported for Aspergilli species).

Machine learning approaches help us gain knowledge from complex patterns in data. Clustering is an unsupervised technique that reveals how instances are naturally grouped in the descriptor space. In clustering, the classes are unknown and are identified by the cluster analysis of the data. In simple terms, the overall idea of clustering is to group similar elements together [16].

**Fig. 2:**
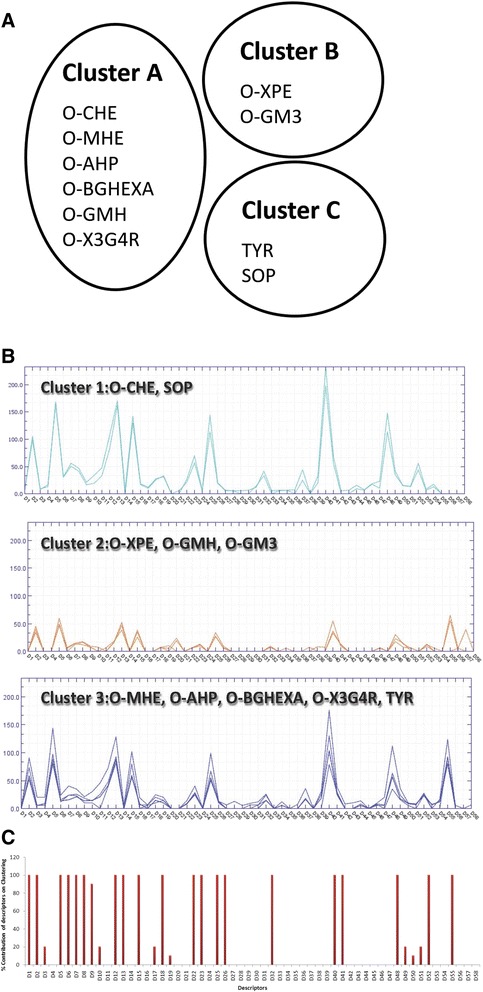
Genome-wide expression signatures of *A. oryzae.* **a** Clustering of the 10 enzyme inducers based on 186 calculated chemical descriptors. *O-CHE* cellohexaose*, O-MHE* mannohexaose*, O-XPE* xylopentaose*, O-AHP,* arabinoheptaose*, O-BGHEXA* 1,3:1,4-β-Glucohexaose*, O-GMH* 6^3^-α-D-Glucosyl-maltotriosyl-maltotriose*, O-GM3* 6^1^-α-D-Galactosyl-mannotriose*, O-X3G4R* xyloglucan (X_3_Glc_4_-Borohydride reduced)*, TYR* turanose*, SOP* sophorose*.* **b** Clustering of the 10 enzyme inducers based on the number of significant genes (in a pair-wise comparison of each oligosaccharide with glucose) per GO term (biological process, cellular component, molecular function). **c** The contribution of each GO term in the clustering is given as a bar chart whereas the names and values of the 58 GO terms are given in Additional file 2

The analysis of the chemical diversity for the above mentioned 10 sugar molecules, based on 186 chemical descriptors calculated from the structure (Additional file 1), resulted into three clusters viz., Cluster A, Cluster B and Cluster C showed in Fig. 2a. The BCUT and GCUT descriptors that consider atomic charge, atomic polarizability-related values, and atomic hydrogen bonding abilities, logP(*o/w*), which is a physical property and the carbon valence connectivity index, showed the highest variance between the 10 enzyme inducers. The oligosaccharides O-XPE, O-GM3 and the di-saccharides TYR, SOP share many of the structural properties and form the distinct clusters 2 and 3 respectively, while the remaining sugar molecules fall into cluster 1.

The pair-wise comparison of the expression of genes between the 10 enzyme inducers and glucose revealed significant differences in expression patterns. O-CHE (2023 genes), O-MHE (1539 genes), O-X3G4R (1723 genes) and SOP (1763 genes) form a group of molecules with the highest number of significant differential expression of genes compared to glucose (*p-*value < 0.05). A second group of molecules, O-AHP, O-BGHEXA and TYR, was found with a moderate number of genes that showed significant changes in gene expression level compared to glucose (880, 1045 and 829 genes, respectively), while O-XPE (568 genes), O-GMH (466 genes) and O-GM3 (451 genes) were the molecules with the lowest number of differentially expressed genes. Using the software Blast2GO [17] and applying the default parameters we obtained the gene ontology (GO) terms of each *A. oryzae* gene whose transcript level differed substantially between the enzyme inducers and glucose. The degree of gene annotation obtained through Blast2GO was in the range of 74–78 % for all the enzyme inducers with the exception of O-X3G4R, which did not exceed the 40 % coverage. In total 58 GO terms were retrieved covering the biological processes (21), cellular components (17) and molecular functions (20). A matrix with the number of significant genes per GO term for each enzyme inducer was constructed (Additional file 2) and used for identifying similar functional patterns between the molecules. The clustering of the chemical inducers based on the above matrix yielded three distinct groups with 2, 3 and 5 members each (Fig. 2b). O-CHE was found to cluster together with SOP, while O-XPE, O-GMH and O-GM3 formed a second cluster whereas the largest cluster included the remaining oligosaccharides. From a parallel look into the clusters generated in Fig. 2a and b we see that only for 6 out of the 10 enzyme inducers the chemically similarity is reflected in the global transcription of the *A. oryzae* genome. The 10-fold cross validation of the GO terms revealed that the following descriptors have the highest contribution towards the clustering of the enzyme inducers (Fig. 2c):

- Biological Process: cellular homeostasis (*D1*), lipid metabolic process (*D2*), catabolic process (*D5*), protein modification process (*D6*), ion transport (*D7*), generation of precursor metabolites and energy (*D8*), multicellular organismal development (*D9*), transcription (*D12*), cellular amino acid and derivative metabolic processes (*D13*), carbohydrate metabolic processes (*D15*), response to stress (*D18*).
- Cellular Component: endoplasmic reticulum (*D22*), nucleoplasm (*D23*), protein complex (*D25*), extracellular region (*D26*), mitochondria (*D32*).
- Molecular Function: nucleotide binding (*D40*), transcription factor activity (*D41*), protein binding (*D48*), electron carrier activity (*D52*), transporter activity (*D55*).

Genes in lipid, protein modification, energy, amino acid and carbohydrate metabolism as well as transcription have been found significantly differentially expressed also in an *A. oryzae* amylase hyper-producer [18].

**Fig. 3:**
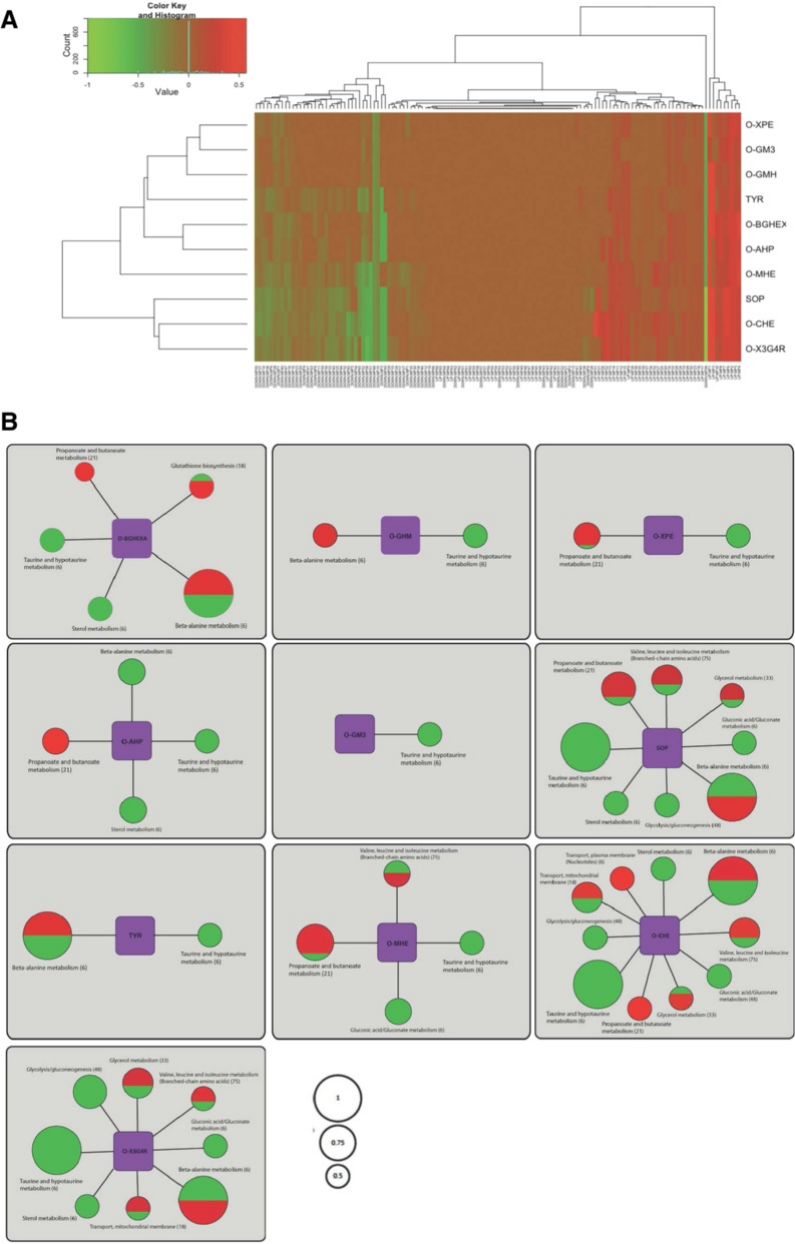
The effects of the different enzyme inducers on the gene expression profile of the metabolic pathways. **a** The ten sugars were clustered based on the fraction of the total genes (that each pathway consists of) that was found significantly different in expression compared to glucose for 66 metabolic pathways. The *green font* indicates down-regulation; the *red font* indicates up-regulation; the values range from 0 (no gene in the pathway found significantly different in expression) to 1 (all the genes in the pathway were found significantly different in expression). The description of the abbreviated pathway names is given in Additional file 3. The clustering tree or the dendogram displayed was based on the heat map of the hierarchical clustering. **b** For each enzyme inducer are given the metabolic pathways that their expression was highly affected (the expression of at least 50 % of the genes involved in the pathway was significantly different compared to glucose). The size of the node indicates the fraction of the pathway up- (*red font*) or down- (*green font*) regulated

To determine the metabolic regulation associated with plant cell wall deconstruction we used the genome scale metabolic network of *A. oryzae* that consists of 2360 reactions [19] and we linked it to the transcript levels of each metabolic gene. O-CHE (365 genes), SOP (323 genes), O-X3G4R (318 genes) and O-MHE (270 genes) samples showed the highest number of metabolic genes with statistically significant difference in relative expression as compared to the glucose sample. In total 196, 185 and 178 metabolic genes were found with significant differences in expression compared to glucose for O-BGHEXA, O-AHP and TYR, respectively, while these numbers were lower for O-XPE (120 genes), O-GM3 (100 genes) and O-GMH (96 genes). In order to get a holistic view of the metabolic differences we evaluated our findings in the context of metabolic pathways instead of the gene level. We first calculated the fraction of the total number of genes in a pathway that was found to be up- and down- regulated in each sample (Additional file 3). Subsequently, we sought to find similarities in the metabolism of the enzyme inducers by clustering them using the above data for the 66 metabolic pathways where transcript levels differed significantly (compared to glucose). Two sets of descriptors for each metabolic pathway were used for the hierarchical clustering analysis; the fraction of up- and the fraction of down- regulated genes (Fig. 3a). The first cluster contains SOP, O-CHE and O-X3G4R, the second cluster contains O-AHP and O-BGHEXA, the third O-XPE, O-GM3, O-GMH and (less similar) TYR, whereas O-MHE seems to have the most unique metabolic regulation pattern (Fig. 3a). Despite a few changes, the clusters formed based on significant differences in the expression patterns of metabolic pathways show high similarity with the clusters obtained previously using the complete GO term annotation (Fig. 2b, c).

Figure 3b gives a visual representation of the metabolic pathways where at least 50 % of the genes in the pathway had a significant difference in expression level (counting both up-regulated and down-regulated genes) between each of the 10 enzyme inducers and glucose. In total, ten such metabolic pathways were found in O-CHE compared to glucose. Taurine and alanine metabolism were the most notable cases. All the genes that are involved in the taurine metabolism were down-regulated in O-CHE compared to glucose. Similarly, the gene signature of alanine metabolism was substantially different between O-CHE and glucose with 3 genes up-regulated and 3 genes down-regulated. All the genes involved in the alanine metabolism were found with significant difference in relative expression compared to glucose also for SOP, TYR, O-X3G4R and O-BGHEXA (Fig. 3b). A different trend regarding the alanine metabolism was observed for O-GMH (3 genes all up-regulated) and O-AHP (3 genes all down-regulated) while no genes were found with significant difference in expression levels in O-GM3, O-MHE and O-XPE compared to glucose. The taurine metabolism was found down-regulated in all the 10 enzyme inducers compared to glucose while the sterol metabolism was down-regulated and the propanoate/butanoate metabolism was (mainly) up-regulated in 6 of them (Fig. 3a). The expression profile of more than 50 % of the genes involved in valine, leucine and isoleucine metabolism was also highly different in SOP, O-CHE, O-MHE and O-X3G4R compared to glucose. An interconnection between cellulase activity and amino acid metabolism has been shown in *T. reesei* [20] and *N. crassa* [21]*,* while Vongsangnak et al. [18] found most of the genes encoding amino acid enzymes to be up- or down-regulated in an amylase *A. oryzae* hyper-producer; our gene expression data support these observations and expand them to other carbohydrate-active enzyme families.

### Secretome analysis of *A. oryzae* gene expression signatures

**Fig. 4:**
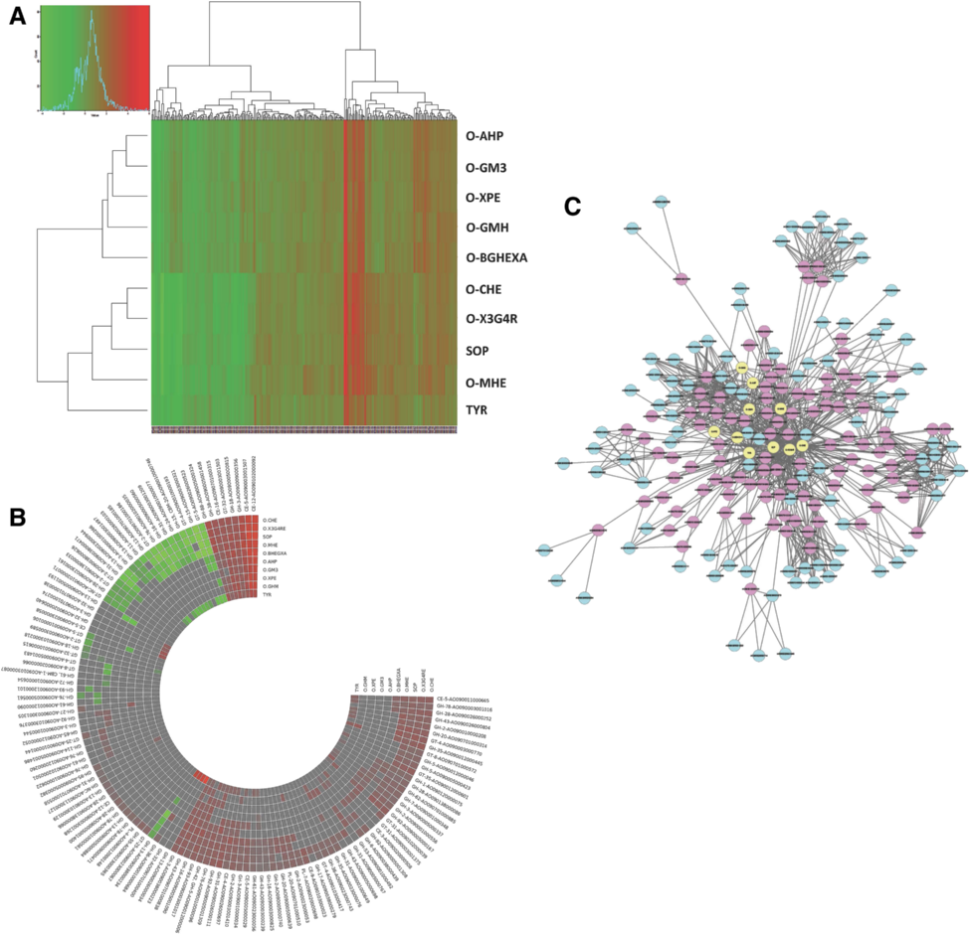
The effects of the different enzyme inducers on the gene expression of extracellular proteins and CAZys. **a** Heatmap of the gene expression profile for genes encoding extracellular proteins with significantly differential expression in the enzyme inducers compared to glucose. The clustering tree or the dendogram displayed was based on the heat map of the hierarchical clustering. **b** Heatmap of the differential gene expression profile of the 124 CAZy transcripts in the enzyme inducers compared to glucose. **c** A network of interactions between genes and inducers; blue nodes are genes encoding CAZys with significant differential expression between the inducers and glucose, red nodes are genes with significant differential expression between induces and glucose that were derived using the ´´guilt-by-association´´ approach, yellow nodes are the individual inducers

The hierarchical clustering analysis of the ten oligosaccharides, based on 263 genes predicted to encode extracellular proteins with significant expression differences in comparison to glucose, is shown in Fig. 4a. According to the continually updated Carbohydrate-Active enZymes (CAZy) database, which is a knowledge-based resource specialized in the enzymes that build and breakdown complex carbohydrates and glycoconjugates [22], *A. oryzae* genome owns 306 translated genes belonging to 56 different Glycoside Hydrolase (GH) families, 119 translated genes belonging to 31 different Glycosyl-Transferase (GT) families, 23 translated genes to 6 different Polysaccharide Lyase (PL) families and 30 translated genes to 9 different Carbohydrate Esterase (CE) families (http://www.cazy.org/e337.html, January 2013). From the 306 translated GH genes, about a third (88 genes) were significantly up- (67 genes) or down- (21 genes) regulated by the presence of one of the 10 oligosaccharides reflecting the potential of these model substrates in the regulation of lignocellulolytic enzymes (Additional file 4). These hydrolases are annotated and are expected to be active in the degradation of various plant cell wall polysaccharides such as cellulose, arabinoxylan and galacto(gluco)mannan (Table 1). Less but also significant was the regulation of carbohydrate biosynthetic enzymes such as GTs, where 22 members were involved (11 up-regulated and 11 down-regulated), 11 up-regulated CEs including one ferulic acid esterase (AO090001000207; http://www.ncbi.nlm.nih.gov/protein/83766610) which is not classified in CAZy database and only 3 up-regulated PLs (Additional file 4). All substrates tested were implicated in the regulation of GH, GT and CEs genes except in case of PLs, where only half of them (O-CHE, O-MHE, O-GMH, O-X3G4R and SOP) were involved, indicating that different structural oligosaccharides are necessary for triggering the pectinolytic system of *A. oryzae*.

**Table 1:** Gene, putative function, CAZy family, induction substrates and significant signal peptide predictions (SignalP 4.0 Server) of major carbohydrate active enzymes transcripts are shown

Gene	Function	CAZy	Induction substrates	SignalP D score
Cellulase				
AO090701000185	Endoglucanase A	GH-12	O-CHE (↓), O-X3G4R (↓), SOP (↓), TYR (↓), O-MHE (↓)	0.604
AO090001000348	Cellobiohydrolase C (CelC)	GH-7	O-CHE (↑), O-X3G4R (↑), O-BGHEXA (↑), O-AHP (↑), SOP (↑), TYR (↑), O-MHE (↑)	0.661
AO090038000439	Cellobiohydrolase	GH-6	O-CHE (↑), O-GMH (↑), O-XPE (↑), SOP (↑)	0.126^a^
AO090001000266	β-glucosidase	GH-3	O-CHE (↓), O-X3G4R (↓), SOP (↓), TYR (↓)	0.696
AO090001000544	β-glucosidase	GH-3	O-MHE (↑)	0.678
AO090701000274	β-glucosidase	GH-3	TYR (↑)	0.865
AO090023000056	Polysacch. monooxygenase	GH-61^b^	O-CHE (↑), O-X3G4R (↑), SOP (↑), TYR (↑), O-MHE (↑)	0.898
AO090103000087	Polysacch. monooxygenase	GH-61^b^, CBM-1	O-CHE (↓), SOP (↓)	0.737
AO090012000090	Polysacch. monooxygenase	GH-61^b^	SOP (↓)	0.653
AO090102000058	Cellobiose dehydrogenase	AA-3	O-CHE (↑), O-X3G4R (↑), SOP (↑), TYR (↑), O-MHE (↑)	0.819
AO090003000346	Aldonolactonase	-	O-CHE (↑), O-X3G4R (↑), O-BGHEXA (↑), O-AHP (↑), SOP (↑)	0.838
Hemicellulase				
Xylan				
AO090026000103	endo-1,4-β-xylanase	GH-11	O-CHE (↓), SOP (↓), O-MHE (↓)	0.874
AO090005000698	β-xylosidase (XylB)	GH-43	O-X3G4R (↑), O-XPE (↑)	0.106
AO090003000239	xylosidase/arabinosidase or endo-1,4-β-xylanase D	GH-43	O-CHE (↑), O-X3G4R (↑), O-AHP (↑), O-GM3 (↑), SOP (↑), TYR (↑), O-MHE (↑)	0.829
AO090001000207	Feruloyl esterase (FAEA)	-	O-CHE (↑), O-BGHEXA (↑), SOP (↑)	0.748
AO090020000508	Acetyl xylan esterase	CE-3	O-BGHEXA (↑), O-GMH (↑)	0.449
AO090701000315	Acetyl esterase	CE-16	O-CHE (↑), O-X3G4R (↑), O-BGHEXA (↑), O-GMH (↑), O-XPE (↑), O-AHP (↑), O-GM3 (↑), SOP (↑), TYR (↑), O-MHE (↑)	0.677
AO090701000885	α-L-arabinofuranosidase (axhA)	GH-62	O-CHE (↑), O-X3G4R (↑), O-BGHEXA (↑), SOP (↑)	0.719
AO090701000838	α-L-arabinofuranosidase	GH-43	O-CHE (↑),O-X3G4R (↑), O-BGHEXA (↑), O-GMH (↑), O-AHP (↑), O-GM3 (↑), SOP (↑), TYR (↑), O-MHE (↑)	0.895
AO090012000445	β-galactosidase (LacA)	GH-35	O-CHE (↑), O-X3G4R (↑), O-GMH (↑), SOP (↑), TYR (↑), O-MHE (↑)	0.713
AO090023000053	β-galactosidase or α-L-arabinofuranosidase B	GH-2	O-CHE (↑), O-X3G4R (↑), SOP (↑), O-MHE (↑)	0.456
Galacto(gluco)mannan				
AO090003001410	β-mannosidase	GH-2	O-CHE (↑),O-X3G4R (↑), O-BGHEXA (↑), O-AHP (↑), O-GMH (↑), O-AHP (↑), SOP (↑), O-MHE (↑)	0.675
AO090001000556	β-mannosidase	GH-2	O-CHE (↑),O-X3G4R (↑), O-BGHEXA (↑), O-AHP (↑), SOP (↑), TYR (↑)	0.492
AO090003001305	α-galactosidase	GH-27	O-CHE (↑), O-GM3 (↑)	0.597
Pectic polysaccharides				
AO090026000252	Rhamnogalacturonase	GH-28	O-CHE (↑),O-X3G4R (↑), O-BGHEXA (↑), SOP (↑), O-MHE (↑)	0.82
AO090003001268	Rhamnogalacturonan acetylesterase	CE-12	O-CHE (↑)	0.804
AO090005001400	Exo-polygalacturonase	GH-28	O-CHE (↑)	0.858
AO090023000161	polygalacturonase	GH-28	O-CHE (↓),O-X3G4R (↓), SOP (↓)	0.531
AO090138000086	endo-polygalacturonase	GH-28	O-CHE (↑), O-X3G4R (↑), O-BGHEXA (↑), SOP (↑), O-MHE (↑)	0.763
AO090003001017	exo-α-L-1,5-arabinanase	GH-93	O-CHE (↑),O-X3G4R (↑), O-BGHEXA (↑), O-AHP (↑), O-GM3 (↑), SOP (↑), TYR (↑), O-MHE (↑)	0.825
AO090026000804	endo-arabinase	GH-43	O-CHE (↑), O-X3G4R (↑), SOP (↑), TYR (↑), O-MHE (↑)	0.81
Other hydrolases				
AO090103000218	Chitinase	GH-18	O-CHE (↓)	0.797
AO090005000029	cutinase (PDB: 3GBS)	CE-5	O-CHE (↑),O-X3G4R (↑), O-AHP (↑), SOP (↑), O-MHE (↑)	0.574
AO090011000665	Cutinase	CE-5	O-CHE (↑),O-X3G4R (↑), O-BGHEXA (↑), SOP (↑), TYR (↑), O-MHE (↑)	0.769
AO090023000058	Cutinase	CE-5	TYR (↑)	0.614

Regulation is indicated by arrows

^a^D-score 0.725 when MHTLNMQALVALSPLLFSAATA is considered as signal peptide omitting 87 residues from the N-terminal

^b^Reclassified recently in CAZy database as AA-9 [25]

We used BLASTp against the NCBI database to annotate the 124 CAZy transcripts in the *A. oryzae* genome that showed a significantly differential expression in the presence of each of the model substrates studied compared to glucose (Fig. 4b and Additional file 4). Among the 88 GH and 10 CE genes, 10 appeared to encode proteins involved, or possibly involved, in β-1,4-xylan hydrolysis (Table 1). These included only three xylan main chain hydrolytic enzymes; one member of family GH-11 endo-1,4-β-xylanase (E.C. 3.2.1.8), the only xylan-degrading down-regulated enzyme in the presence of O-CHE, SOP and O-MHE compounds, and two GH-43 β-xylosidases (E.C. 3.2.1.37). The XylB (AO090005000698) was found to be up-regulated only in the presence of the xylose containing oligosaccharides O-X3G4R and O-XPE, however, the AO090003000239 β-xylosidase gene might express endo-1,4-β-xylanase activity due to homology with *Bacteroides thetaiotaomicron* xylanase D (crystal structure; PDB 3QZ4). Moreover, seven hydrolases that cleave xylan decorations include extracellular accessory enzymes such as two α-L-arabinofuranosidases (E.C. 3.2.1.55) members of GH families 43 and 62, one β-galactosidase (E.C. 3.2.1.23), which is homologous to *A. niger* LacA, and three esterases including a ferulic acid esterase (E.C. 3.1.1.73) not classified in CAZy database and two acetyl esterases (E.C. 3.1.1.72) members of CE-3 and the recently discovered CE-16 families. One more xylan auxiliary GH-2 enzyme product of the gene AO090023000053 induced by O-CHE, O-X3G4R, SOP and O-MHE, possibly exhibit β-galactosidase or α-L-arabinofuranosidase B activity due to high homology with characterized members of both enzyme classes. The major induction of accessory enzymes instead of xylan main chain hydrolases may be explained due to the small degree of polymerization (DP) of the model oligosaccharides tested, which could be *A. oryzae* hydrolytic products of main chain hydrolases. In most cases, the xylose containing oligosaccharide O-X3G4R was found as a common inducer of xylanolytic activities, in contrast to O-XPE substrate that induced only XylB xylosidase and acetyl esterase genes. However, glucose containing substrates, such as O-CHE seems to trigger xylanolytic activities indicating a common induction mechanism involved.

In the presence of various xylose or glucose containing oligosaccharides, the *A. oryzae* transcriptome exhibited the up-regulation of few galacto(gluco)mannan accessory hydrolases including two β-mannosidases (E.C. 3.2.1.25) members of GH-2 family and one α-galactosidase (E.C. 3.2.1.22) member of GH-27 family (Table 1). The small number of galactomannan degradation enzymes is probably related to the absence of galactoside or mannoside residues in the induction molecules used, except in the cases of O-MHE and O-GM3. O-MHE is one of eight model substrates that trigger one GH-2 β-mannosidase, while O-GM3 seems to be more important for the induction of α-galactosidase, as together with O-CHE are the only substrates that induced this accessory enzyme. All aforementioned hemicellulolytic accessory enzymes release L-arabinose, D-galactose, D-mannose, acetic and ferulic acid from the β-1,4-xylan or galacto(gluco)mannan main chains, and were all predicted to be extracellular. The only exception is AO090005000698 β-xylosidase which might not function outside the cell environment, since the ortholog enzyme from *Penicillium herquei* does not possess a signal peptide but is localized in the cell surface [23].

Cellulose is degraded by the coordinated action of endoglucanases (E.C. 3.2.1.4) and cellobiohydrolases (E.C. 3.2.1.91), while cellobiose and small cellooligosaccharides are hydrolyzed by β-glucosidases (E.C. 3.2.1.21) [24]. Four genes translating most of the above activities were up-regulated exhibiting two cellobiohydrolases members of GH families 6 and 7 and two β-glucosidases members of GH family 3 (AO090001000544 and AO090701000274), while two genes were down-regulated including one GH-12 endoglucanase A and one GH-3 β-glucosidase (AO090001000266; Table 1 and Additional file 4). Recent transcriptomic and proteomic analyses of cellulolytic fungi have identified oxidative enzymes involved in degradation of plant biomass [25, 26]. Fungal-derived, copper-dependent polysaccharide monooxygenases (PMOs) or lytic polysaccharide monooxygenases (LPMOs), formerly known as weak endoglucanase GH-61 proteins, have been recently shown to catalyze the O_2_-dependent oxidative cleavage of recalcitrant polysaccharides [27]. In addition, it has been shown a synergistic effect of both *Thielavia terrestris* GH-61 and cellobiose dehydrogenase (CDH; EC 1.1.99.18) on microcrystalline cellulose hydrolysis by *T. terrestris* canonical cellulases [28]. Currently, there are more than 200 CAZy entries for GH family 61, which is now reclassified in the new established family of Auxiliary Activity (AA) 9 [29], all from eukaryotic sources, with *A. oryzae* genome to contain eight members. Two of the three PMOs were down-regulated while AO090023000056 was up-regulated in the presence of various model substrates, having in common SOP. An *A. oryzae* homologue (AO090102000058) to *Myceliophthora thermophila* CDH (protein ID 58125; 61.3 % identity), member of the CAZy AA family 3, was up-regulated in the presence of O-CHE, O-X3G4R, SOP, TYR, O-MHE, enhancing cellulose degradation by coupling the oxidation of cellobiose to the reductive activation of copper-dependent PMOs that catalyze the insertion of oxygen into C − H bonds adjacent to the glycosidic linkage [30]. Interestingly, an extracellular aldonolactonase homologue to two recently discovered *M. thermophila* aldonolactonases (31.2 % identity with protein ID 89286 and 29.7 % identity with protein ID 109678) was up-regulated in the presence of O-CHE, O-X3G4R, O-BGHEXA, O-AHP and SOP, an enzyme with possibly a very important role in cellulose utilization. These recently discovered enzymes catalyze the hydrolysis of glucono-δ-lactone and cellobiono-δ-lactone that are produced by the enzymatic oxidation of cellulose [27]. Sugar lactones have been shown to inhibit many different types of glycosyl hydrolases [31]. For example, glucono-δ-lactone is a potent inhibitor of β-glucosidases [32]. All up-regulated cellulases reported except β-glucosidases AO090001000544 and AO090701000274, indicated SOP as a common inducer. SOP is a well known cellulase inducer that was originally isolated from culture fluids of *Hypocrea jecorina* [33] and has been shown to be produced during growth on cellobiose as carbon source [34] or after cellulose hydrolysis and transglycosylation mediated by β-glucosidases acting in concert with the fungal cellulase system [35].

Interestingly, in the presence of the ten model inducers, *A. oryzae*’s cell wall degrading system up-regulated also hydrolases involved in pecticolysis. These enzymes act mainly on the pectin backbone composed of homogalacturonan by the action of one endo-polygalacturonase (E.C. 3.2.1.15) and one exo-polygalacturonase (E.C. 3.2.1.67), which are members of the GH family 28, and rhamnogalacturonan, which is degraded by a GH-28 rhamnogalacturonase (E.C. 3.2.1.171) and a CE-12 rhamnogalacturonan acetyl esterase (E.C. 3.1.1.-) (Table 1). Polygalacturonase AO090023000161, a member of the GH family 28, was down-regulated in the presence of three oligosaccharides, viz., O-CHE, O-X3G4R, SOP, indicating possibly the different role of this isoenzyme compared to other pectin hydrolyzing enzymes. In the primary cell wall, pectins are characterized by a high quantity of neutral sugar side chains, such as arabinan and galactan. The *A. oryzae* transcriptome revealed two arabinan-acting enzymes such as an exo-α-L-1,5-arabinanase (E.C. 3.2.1.-) member of the GH family 93 and an endo-arabinanase (E.C. 3.2.1.99) member of the GH family 43. All putative pectin-modifying enzymes were predicted to be extracellular and up-regulated in the presence of different model substrates having in common the inducers O-CHE, O-X3G4R and SOP. However, a GH-28 exo-polygalacturonase was induced by the presence of solely O-CHE.

In the need of identifying novel enzymatic systems that will allow us to expand our knowledge in plant biomass utilization, a “guilt-by-association” approach was applied to identify novel genes/proteins involved in the degradation of lignocellulosic biomass (Fig. 4c; Additional file 5). Under this view, 90 genes that their expression products are predicted to be secreted to the extracellular space and follow the same expression profile in a particular set of substrates as does a specific CAZy member, are leading us indirectly to a possible enzymatic involvement to this CAZy members. Following this approach, a number of up-regulated peptidases that are predicted to be directed to the secretory pathway are correlated with the expression profile of various CAZy genes, e.g. arabinogalactan endo-1,4-β-galactosidases (AO090102000079; serine carboxypeptidase) and endo-1,3(4)-β-glucanase or β-galactosidase (AO090020000517; subtilisin-like serine protease). Saprotrophic fungi is well known in secreting peptidases in order to degrade a variety of (poly)peptides in their environment for utilizing nutrients or eliminate antifungal host proteins. The necrotrophic fungal pathogens *Sclerotinia sclerotiorum* and *Botrytis cinerea* were analyzed for genes encoding proteins with predicted proteolytic activity, with emphasis on the secreted peptidases, showing a variety of peptidases comparable to the *Eurotiomycetes* and *Sordariomycetes* [36]. *S. sclerotiorum* and *B. cinerea* possess a large number of genes encoding peptidases with an acidic pH optimum suggesting an adaptation of the peptidase gene content to perform well in a low pH environment generated by the production of oxalic acid. It has been also shown that proteases play an important role in solubilizing brewer’s spent grain, enhancing ferulic acid release from the material treated with protease prior to enzymatic hydrolysis [37]. In addition to peptidases, other non-CAZy genes that are involved in cell wall decomposition were recovered using the ´´guilt-by-association´´ approach [38], such as cytochrome P450 monoxygenases (AO090023000072 down-regulated and AO090011000712 up-regulated) related to β-glucosidases, cellobiohydrolases or various glucanases (Additional file 5). Various monooxygenases have been evolved in the degradation of small lignin fragments and other aromatic compounds facilitating growth on softwood substrates by detoxifying lignin degradation products and the higher extractive content than is present in most hardwood species [39]. In order to evaluate the role of the aforementioned regulated genes in plant biomass degradation, the cloning and characterization of the corresponding enzymes is necessary.

### Interplay of metabolites and transcription factors on the modulation of genes encoding plant polysaccharide degrading enzymes

The list of the 124 CAZy enzymes induced in the presence of the 10 chemically defined model substrates underpins the complexity of the regulatory mechanisms. The genes encoding fungal plant polysaccharide degrading enzymes are regulated by several transcription factors; the xylanolytic transcriptional activator XlnR involved in controlling the expression of genes involved in cellulose, xylan, xyloglucan and galactomannan degradation [40–42], the amylolytic transcriptional activator AmyR involved in the regulation of genes involved in starch degradation [43], the carbon catabolite repressor CreA repressing the expression of genes encoding plant polysaccharide degrading enzymes in the presence of other carbon sources [44]. Given the fact that the cell’s functional proteins (e.g. enzymes) and regulatory proteins (e.g. transcription factors) are bathed in a pool of metabolites, it is reasonable to speculate that their structure and function can be modulated by interacting not only with their substrates or ligands, but also by the metabolites [45]. Our effort here to elucidate the interplay of metabolites and transcription factors, using the 814 unique reactants involved in 2360 reactions in the genome scale metabolic network of *A. oryzae* [46], required the three-dimensional protein structures of the aforementioned transcription factors; hence several homology-model structures were derived using *ab-initio multiple threading alignment* (see details in Additional files 6 and 7). The Protein Data Bank (PDB) structures used for multi template modeling and their respective secondary structure alignments are given in the Additional file 6. The *ab-initio multiple threading alignment* approach [47–49] was based on the I-TASSER predictor followed by LOMETS threading. The modeled structures of the three *A. oryzae* transcription factors had a C-score in the range of (-5 and 2), which signifies a model with a high confidence [47, 50, 51]. The TM-scores of the modeled transcription factors viz., XlnR, AmyR and CreA were 0.54, 0.61 and 0.62 respectively, with a TM-score >0.5 to indicate a model of correct topology. TM-score is a recently proposed scale for measuring the structural similarity between two structures [52] with the intension to solve the problem of RMSD, which is sensitive to the local error. Furthermore, the verification scores viz., DOPE (Discrete Optimized Protein Energy) score, DOPE-HR (High Resolution) score, Verify score and the potential energies of the modeled structures (Additional file 6) revealed high quality of the generated models. Finally, the CHARMM based Energy minimization of the modeled structures was performed to remove steric overlaps that cause bad contacts; the initial potential energies of starting structures and the potential energy of respective minimized structures are given in Additional file 6. The coordinates of the model structures with high C-score and TM-score were submitted to the Protein Model DataBase (PMBD: http://bioinformatics.cineca.it/PMDB/) [53]. This high quality refined model structures are the ones we used for analysis of the binding pockets and docking studies.

**Fig. 5:**
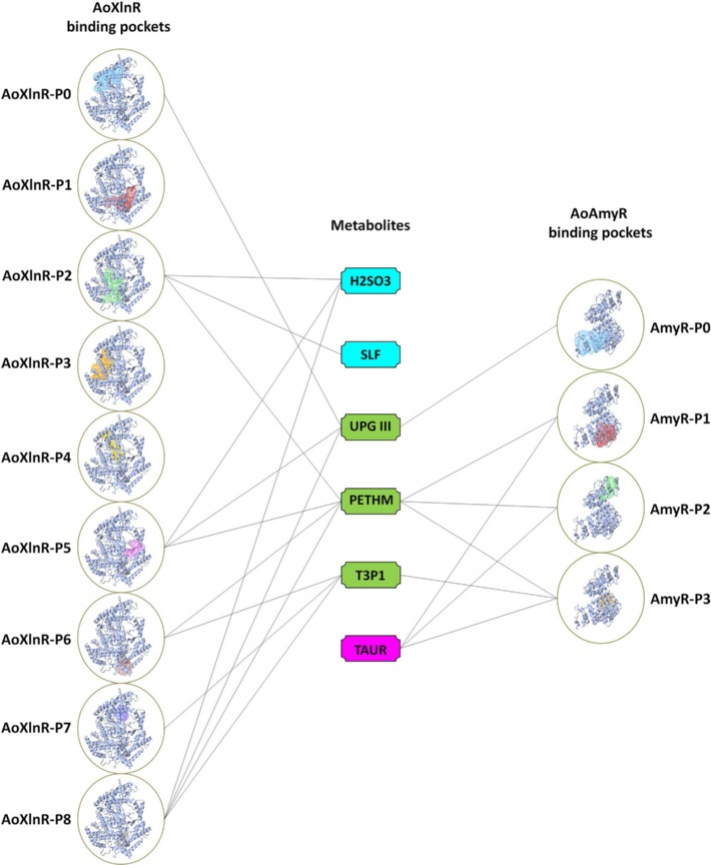
The binding pockets (P0, P1, P2…) of AoXlnR and AoAmyR and the proposed interaction map of their strong binding metabolites viz., sulfite (H2SO3), sulfate (SLF), uroporphyrinogen III (UPGIII), ethanolamine phosphate (PETHM), D-glyceraldehyde 3-phosphate (T3P1) and taurine (TAUR). AoXlnR and AoAmyR are represented as ribbon structures. The binding pockets of AoXlnR and AoAmyR are rendered as solvent surface representations. For clarity, the location of binding pockets P7 and P8 of AoXlnR are shown with *small arrows* inside the respective *circle*. The metabolites that strongly bind to the binding pockets of AoXlnR only are *highlighted in blue*, whereas the metabolites that strongly bind to the binding pockets of AoAmyR only are *highlighted in blue magenta*. The common metabolites i.e, the metabolites that strongly bind to the binding pockets of both AoXlnR and AoAmyR are *highlighted in green*. The *lines* between the metabolites and the binding pockets indicates that respective metabolites can bind to respective binding pockets

Subsequently, we attempted to identify the strong metabolite binders of the transcription factors AoXlnR and AoAmyR and their impact on the process of enzyme regulation for plant polysaccharide degradation in *A. oryzae*. The binding pockets of AoXlnR and AoAmyR were predicted using a structure-based method relying on the Difference of Gaussian (DoG) approach [54]. In this study, the binding pockets with a score ≥ 0.8 were selected for molecular docking. As shown in Additional file 8, the structure of AoXlnR possess a total of 9 binding pockets, whereas the structure of AoAmyR possess a total of 4 binding pockets with scores ≥ 0.8. Systematic investigation of metabolite-protein interactions was performed through a two-step molecular docking of the entire metabolome of *A. oryzae* against the binding pockets of AoXlnR and AoAmyR. In the first step, the 814 metabolites were docked into each of the binding pockets using the LibDock algorithm [55–58] to filter binders from non-binders. In the second step, the strong binders were shortlisted using the HYDE scoring function [59]. The advantage of using HYDE scoring is the heavy penalization of unmet interactions: A hydrogen bond taken out of the solvent - and not having an ideal partner in the protein; or the phenyl ring that is dehydrated and put into a hydrophilic active site region. Thus, not by rewarding H-bonds but by penalizing unfavorable situations, false positives are effectively ruled out in an *in silico* screen [60]. A total of six metabolites viz., sulfite (H_2_SO_3_), sulfate (SLF), uroporphyrinogen III (UPGIII), ethanolamine phosphate (PETHM), D-glyceraldehyde 3-phosphate (T3P1) and taurine (TAUR) were found as strong binders. The strong binding metabolites of the respective AoXlnR and AoAmyR binding pockets are shown in Fig. 5.

It is intriguing to observe that both AoXlnR and AoAmyR share three strong binding metabolites UPG III, PETHM and T3P1. The metabolites H_2_SO_3_ and SLF are specific to the binding pockets of AoXlnR; whereas TAUR is the only metabolite that specifically binds to AoAmyR. The levels of these strong binding metabolites depend on the turnover of the reactions in the cell. Binding of single or multiple metabolites to the binding pockets of the transcription factors may change their protein conformations, which in turn modulate the transcription factor binding to the regulatory regions of the DNA. Furthermore the degradation of complex plant biomass requires the expression of a cocktail of enzymes by *A. oryzae* to efficiently assimilate the degradation products of plant polysaccharides. It is rare to find plant polysaccharides independently in a habitat and the presence of complex polysaccharides require the coordinated expression of enzymes that can efficiently degrade them. A recent study on mapping of polysaccharide degradation potential of *A. niger* by Andersen et al. [61] have shown that even though there are no similar bond types in starch and xylan, the gene expression analysis of cellulases and hemicellulases coding genes in *A. niger* cultures grown on these two substrates indicated the opposite. This is in line with our observations in the previous section regarding differential expression of genes in *A. oryzae* cultures induced by different chemically defined substrates. As shown in Table 2, it is interesting to observe that the logFC values of respective genes follow the same trend in the *A. oryzae* cultures (i.e., the genes associated with UPG III, H_2_SO_3_, SLF and TAUR are down-regulated in the presence of different inducers compared to the gene expression pattern in the presence of glucose, whereas the genes associated with PETHM and T3P1 are up-regulated), which supports our speculation that metabolites may modulate the regulatory networks of enzyme expression by influencing the conformations of transcription factors. As regulators of the transcriptional machinery of plant polysaccharide degrading enzymes, metabolites indirectly can exert a global effect on the cell’s metabolism.

**Table 2:** Associations between the metabolites-reactions-enzymes-genes-logFC values. Positive logFC values indicate the logarithmic foldness of up-regulation of respective genes

Metabolite	Metabolite's Reaction	Enzyme catalyzing the reaction	Gene association of the enzyme	logFC compared to Glucose									
				O-BGHEXA	O-GMH	O-XPE	O-AHP	O-GM3	SOP	TYR	O-MHE	O-CHE	*X*_*3*_ *Glc*_*4*_
H_2_SO_3_	H_2_SO_3_ + 3 NADPH => H_2_S + 3 NADP + 3 H_2_O	Sulfite reductase (NADPH)	AO090001000571	−1.74	−2.52	−2.07	−2.24	−2.16	−4.11	−2.5	−3.51	−4.4	−4.61
SLF	SLFe => SLF	Sulfate transporter	AO090003000798	−1.53	−1.52	-	−1.74	−1.56	−4.26	−3.57	−4.12	−3.75	−4.08
UPGIII	SAM + UPGIII => SAH + PRECOR	Uroporphyrin-III C-methyltransferase	AO090020000339	−1.57	−1.82	−1.5	−1.57	−1.37	−2.44	−2.36	−3.18	−2.48	−2.64
PETHM	PE => DAGLY + PETHM	Phospholipase C	AO090103000073	1.26	1.28	1.1	0.95	0.75	1.04	0.91	1.26	0.95	0.74
T3P1	R5P + XUL5P <=> S7P + T3P1	Transketolase	AO090023000345	1.2	1.09	1.04	1.27		1.08	0.98		1.33	1.27
	E4P + XUL5P <=> F6P + T3P1							-			-		
	XUL5P + FALD <=> T3P1 + GLYN												
TAUR	TAUR + AKG + O_2_ => H_2_S + AMIACE + SUCC + CO_2_	Alpha-ketoglutarate-dependent taurine dioxygenase	AO090023000531	−1.4	-	−1.69	−1.55	−1.44	-	−1.53	−2.1	−1.86	-

Negative logFC values indicate the logarithmic foldness of down-regulation of respective genes

*Abbreviations*: *H*_*2*_ *SO*_*3*_ sulfite, *SLF* sulfate, *UPGIII* Uroporphyrinogen III, *PETHM* ethanolamine phosphate, *T3P1* D-glyceraldehyde 3-phosphate, *TAUR* taurine, *O-BGHEXA* 1,3:1,4-β-glucohexaose, *O-GMH* 6^3^-α-D-glucosyl-maltotriosyl-maltotriose, *O-XPE* xylopentaose, *O-AHP* arabinoheptaose, *O-GM3* 6^1^-α-D-galactosyl-mannotriose, *SOP* sophorose, *TYR* turanose, *O-MHE* mannohexaose, *O-CHE* cellohexaose, *X*_*3*_ *Glc*_*4*_ *-borohydride reduced; O-X3G4R* xyloglucan

#### Network analysis of the proteins involved in the metabolic reactions of strong binding metabolites

Enthused by the observation that a handful of metabolites can strongly bind to AoXlnR and AoAmyR, we analyzed the chemical-protein interaction networks of the enzymes that catalyze the reactions in which the strong binding metabolites of AoXlnR and AoAmyR are involved either as a substrate or product. It is especially interesting to observe that there is an inter-regulation between the metabolites that bind to the transcription factors and enzymes that catalyze the reactions in which they are involved. The chemical-protein interaction network (shown in Additional file 7: Figure S7) depicts the interplay of the metabolite UPG III and the enzyme UPG-III methyltransferase. In addition, the chemical-protein interaction network shows that there is a relation between the UPG-III methyltransferase and sulfite reductase, which is the enzyme connected to the sulfite (metabolite) that can strongly bind to AoXlnR but not to AoAmyR. These observations show the complex cross-talk between the regulators of the polysaccharide degrading enzyme regulators.

To understand the chemical-protein interaction network shown in Additional file 7: Figure S7, we did literature mining to find experimental evidence of cross-talk between the sulfite metabolism and their effects on the gene expression in microorganisms. We found recent experimental studies demonstrating the sulfite can affect the gene expression patterns in plants, yeast and fungi [62–64]. Plants utilize the inorganic sulphate from soil, which is further reduced to sulfite (reaction catalyzed by sulfite reductase) and assimilated into organic compounds. Plants can also assimilate atmospheric sulfur and it has been shown that sulfur-rich compounds/sulfites enhance the biotic defense mechanisms in plants [65]. Experimental studies by Gramel et al [64], have shown that sulfur metabolism and cellulase gene expression in filamentous fungi are connected processes, but they were not able to find the molecular relationship between the two processes.

In addition, sulfite is considered toxic to many microorganisms even though the mode of action is not clearly understood [64, 66, 67]. So playing the devil's advocate, a scenario could be that plants use sulfur-rich metabolite interaction networks for defense responses where microorganisms utilize the same metabolite as a signal to activate the transcriptional regulator of the hydrolytic enzymes to degrade the plant polysaccharides. Since the task of regulation is highly condition-specific and depends on the interaction of several players, empirical assessment of the number of players and conditions would be an infinite task. Our approach to understand the molecular basis of influencing the regulators of plant polysaccharide-degrading enzymes represents a search to capture the patterns that can be applied to regulatory systems and networks in living cells in general. The conceptual shift in our understanding of metabolites may provide more evidence in future on their role in regulatory circuits that govern the cellular operations.

## Conclusions

Due to the complexity of metabolizing plant biomass, we used in this study a systems-level strategy combining genome-wide transcriptome analysis, protein modeling and high-throughput molecular docking to increase our understanding on the fine-tuning of the plant cell wall degrading enzyme repertoire of *A. oryzae*. We showed that *A. oryzae* alters the expression level of more than 120 CAZys as well as diverse metabolic processes in response to enzyme inducers creating a sophisticated interaction network that may help the fungi survive if conditions and substrate availability change. The expression of set of enzymes that are not needed, based on the chemical features of the chemical inducers used in our experiments, could be interpreted as a way to reduce the delay inherent in adaptive control. However, even though the data presented here provide a blueprint indicating some interesting genetic targets and regulatory elements, we need to collect additional evidence for engineering microbes to efficiently deconstruct plant biomass.

### Limitations and future work

There are several transcriptome analysis studies that allowed the incubation of the target microorganism in the presence of inducing compounds through a short (couple of hours) [13, 14, 68–71] up to a long (24 or even 50 h) time frame [72, 73]. In the latter studies the inducer was treated, as carbon source where extended time is required for its consumption. In this study, the inducer is simply the signaling molecule that will trigger the response from the fungus that has been previously grown on glucose. Furthermore, we were interested in monitoring the fast responsive carbohydrate active enzymes and metabolic networks regulated by the presence of the ten model substrates during the short two-hour incubation. A longer cultivation time together with the low final concentrations of the inducer (in the range of mg/L) used in our experiments would put us in the risk of complete consumption of the oligosaccharides and subsequently missing the respective gene expression response. However, it will be interesting to see the pattern of cellulases and hemicellulases gene expression under different induction times.

Based on our *in silico* analysis, six metabolites viz., sulfite (H_2_SO_3_), sulfate (SLF), uroporphyrinogen III (UPGIII), ethanolamine phosphate (PETHM), D-glyceraldehyde 3-phosphate (T3P1) and taurine (TAUR) were found as strong binders of the transcription factors AoXlnR and AoAmyR. Among the proposals for future developments based on the present study, experimetal design and verification should be included in connection with the impact of the above mentioned six metabolites on the process of enzyme regulation for plant polysaccharide degradation in Aspergillus species.

## Methods

### Strains

The strain used in this study was the *A. oryzae* sequenced strain RIB40, obtained from the IBT culture collection at the Technical University of Denmark. The strain was maintained as frozen spore suspensions at -80 °C in 20 % glycerol.

### Growth medium

The composition of the batch cultivation medium was the following (in g L^−1^): 20 g glucose monohydrate, 2.0 g MgSO_4_.7H_2_O, 2 g K_2_SO_4_, 2.0 g KH_2_PO_4_, 5.0 g (NH_4_)_2_SO_4_, 0.5 ml L^−1^ pluronic acid (PE-6100) (BASF SE, Ludwigshafen, Germany) and 0.6 ml L^−1^ of trace elements solution. Trace elements solution composition (in g L^−1^): 14.3 g ZnSO_4_.7H_2_O, 8.5 g MnSO_4_.H_2_O, 13.8 g FeSO_4_.7H_2_O, 2.5 g CuSO_4_.5H_2_O, 3 g citric acid monohydrate (as a chelating agent) and 0.5 g NiCl_2_.6H_2_O.

*A. oryzae* spore propagation medium (Cove-N-Gly) (in g L^−1^): 218 g sorbitol, 10 g glycerol 99.5 %, 2.02 g KNO_3_, 25 g agar and 50 ml L^−1^ of salt solution. Cove-N-Gly salt solution (in g L^−1^): 26 g KCl, 26 g MgSO_4_.7H_2_O, 76 g KH_2_PO_4_ and 50 ml L^−1^ of trace elements solution. Cove-N-Gly trace elements solution (in mg L^−1^): 40 mg Na_2_B_4_O_7_.10H_2_O, 400 mg CuSO_4_.5H_2_O, 1200 mg FeSO_4_.7H_2_O, 700 mg MnSO_4_.H_2_O, 800 mg Na_2_MoO_4_.2H_2_O and 10 mg ZnSO_4_.7H_2_O.

### Batch fermentations

*A. oryzae* RIB40 batch fermentations were inoculated with spores propagated on Cove-N-Gly spore propagation medium plates incubated for 5 days at 30 °C. To obtain enough mycelia with well dispersed filamentous growth grown under the same conditions to start enzyme induction with ten different protein inducing oligosaccharides in 250 mL shake flask cultivations, two consecutive batch cultivations were carried out. *A. oryzae* batch fermentations were performed in 3.6 L Infors bioreactors with a working volume of 2.8 L. Reactors were equipped with two Rushton four-blade disc turbines, no baffles, pH and temperature control. The temperature was maintained at 30 °C and the pH was controlled by automatic addition of by addition of 10 % H3PO4 or 10 % NH3 solution. The pH was set to 3.5 to prevent spore aggregation and kept constant throughout the cultivation. The stirring speed was initially set at 200 rpm and the aeration rate to 0.1 vvm (volume of gas per volume of liquid per minute). After germination, these parameters were increased to 1000 rpm and 1 vvm and kept steady throughout all the rest of the fermentation.

The batch cultivation was used to harvest biomass for consecutive induction by any of the different oligosaccharides at the final concentration of 200 mg L^−1^. Unless otherwise stated, the oligosaccharides used were obtained from Megazyme (Megazyme, International Ireland Ltd, Wicklow, Ireland); solely sophorose, D-melibiose and D-turanose were obtained from Sigma (Sigma-Aldrich Inc., St. Louis, MO, United States). Oligosaccharides were very well dissolved in water, filtered sterilized and added to each of the three shake flask replicates.

Mycelium was harvested after ~32 h cultivation in mid-exponential phase. Mycelium was washed with sterile mineral media without carbon source, squeezed and weighed under sterile conditions. 800 mg of wet weighed mycelia were used for inoculation of each shake flask cultivation.

### Total RNA extraction

For gene expression analysis, mycelium was harvested after 2 h cultivation with the respective inducer from each of the three biological replicates. Several recent reports employs similar incubation time duration chosen in this study [68–70]. The cultures were filtered through sterile Miracloth (Calbiochem, San Diego, CA, USA) and washed with a suitable amount of 0.9 % NaCl solution. The mycelium was quickly dried by squeezing and subsequently frozen in liquid nitrogen. Samples were stored at -80 °C until RNA extraction. *A. oryzae* total RNA was extracted using the Qiagen RNeasy Mini Kit (QIAGEN Nordic, Ballerup, Denmark), according to the protocol for isolation of total RNA from plant and fungi. For the purpose, approximately 500 mg of frozen mycelium was ground to powder using a ceramic mortar and pestle. Mycelium was kept in liquid nitrogen throughout the grinding processing. All samples were inspected for good quality of total RNA extracted with a BioAnalyzer (2100 BioAnalyzer, Agilent Technologies Inc., Santa Clara, CA, USA). RNA quantification was performed in a spectrophotometer (Biophotometer 6131, Eppendorf AG, Hamburg, Germany) and total RNA was stored at -80 ^o^C until further processing.

### Microarray manufacturing and design

Custom-designed NimbleGen microarrays were used for the analysis of the transcriptome data of *A. oryzae* (Roche NimbleGen Inc., Madison, WI, USA). The arrays used were 4X72K format containing 72,000 60mer probes targeting each of the four individual samples hybridized and analyzed simultaneously on each array. In general, for this kind of arrays transcripts have ≥ 3 probes per gene with a 3’-ORF-based preference. Arrays were processed with reagents according to the NimbleGen Arrays User’s Guide version 5.0 in the Instrument System suggested. The design and selection of probes for interrogating gene expression levels based on the genome of *A. oryzae* RIB40 (DOGAN database) was performed by the Company and contained 12,039 probe sets covering the *A. oryzae* genome plus an EST collection (courtesy of Novozymes).

### Preparation of Cy3-labeled cDNA and microarray processing

Cy3-labeled cDNA was prepared from total RNA, according to the protocol described in the NimbleGen Arrays User’s Guide version 5.0 (Roche NimbleGen Inc., Madison, WI, USA). All samples were prepared in the same manner. Cy3-labeled cRNA was quantified in a spectrophotometer (Amersham Pharmacia Biotech, GE Healthcare Bio-Sciences AB, Uppsala, Sweden) and was fragmented following the manufacturer recommendations. Fragmented cDNA was hybridized to the NimbleGen *A. oryzae* custom-designed array following the manufacturer recommendations. Arrays were washed and stained using a GeneChip® Fluidics Station FS-400, and scanned on an Agilent GeneArray® Scanner 3000. The scanned probe array images were converted into data files using the NimbleScan software.

### Computational tools and databases

#### Descriptor calculations

The SMILES of the 10 substrates were imported into MOE where they were washed and protonated and the 3D structures of the substrates were computed. All 186 2D descriptors were calculated in MOE and the data were exported to Orange (http://orange.biolab.si) where a 5x5 rectangular self-organized map (SOM) was trained.

#### Clustering

The clustering [16] of the sugars based on the number of significant genes (in a pair-wise comparison with glucose) per GO term was performed using J-Express v1.3 (J-Express, Molmine AS, Norway, http://www.molmine.com/). An intermediate between clustering and multidimensional scaling is provided by J-Express [74] through the implementation of a Self-Organizing Map (SOM) algorithm. J-Express allows the user to intitate *k*-means clustering from the clusters resulted by SOM, which overcomes the problem of having to specify the number of clusters in traditional *k*-means clustering. The approach of *k*-means clustering of SOM has been successfully employed for the identification of distinct gene expression patterns [75]. For the training of SOM, we used a starting input grid of 24 neurons and 4000 iterations, while the Gauss neighborhood function [76] and the Euclidean distance measure [77] were applied for updating the grid. Subsequently, the output of SOM was fed as input vector to the *k*-means clustering algorithm, in order to define the boarders between the nodes and to put in the same cluster nodes that were close to each other. For consistency, we used again 4000 iterations and Euclidean distance measure. The variation in the values among the 58 descriptors was high, so we standardized the data and further used the attribute evaluator method [78] that evaluates the importance of a subset of descriptors by considering the individual predictive ability of each one along the degree of redundancy between the descriptors.

#### Metabolic pathways

The *A. oryzae* metabolic model developed by Vongsangnak et al [19] was downloaded from the BioMet Toolbox of Prof. Nielsen’s lab (http://biomet-toolbox.org/index.php?page=models-A.oryzae). The metabolic reactions and the corresponding gene names were loaded into a Perl hash and the significant genes for each substrate were linked to the corresponding metabolic pathway. The chemical-protein interaction networks were extracted using STITCH 3.1, which is a resource to explore known and predicted interactions of chemicals and proteins [79].

#### Manual annotation

Genes that are induced in the presence of the ten model oligosaccharides and translated to carbohydrate active enzymes were manually annotated. Translated amino acid sequences of gene models obtained at DOGAN were subjected to BLASTP homology search at the National Center for Biotechnology Information (NCBI; http://blast.ncbi.nlm.nih.gov/Blast.cgi) to identify homologous proteins with experimentally determined function. Members of the GH, GT, PL and CE families were classified according to the CAZy database (http://afmb.cnrs-mrs.fr/CAZY/). The existence of signal peptide sequences were predicted using SignalP v4.0 server [80].

#### Protein modeling and molecular simulations

Three-dimensional atomic models for the *A. oryzae* transcription factors viz., Xylanolytic transcriptional activator XlnR, Amylolytic transcriptional activator AmyR and Carbon catabolite repressor CreA were modeled from multiple threading alignments [81] and iterative structural assembly simulations using I-TASSER algorithm [47, 48, 50, 51]. Structure refinement of modeled structures was carried out using the Discovery Studio software suite version 3.0 (Accelrys Inc, USA). The Prepare Protein protocol package in Discovery Studio suite was used for inserting missing atoms in incomplete residues, modeling missing loop regions [82], deleting alternate conformations (disorder), standardizing atom names, and protonating titratable residues using predicted pKs [83]. The Side-Chain Refinement protocol was used for each structure to optimize the protein side-chain conformation based on systematic searching of side-chain conformation and CHARMM Polar H energy minimization [84] using the ChiRotor algorithm [85]. Smart Minimizer algorithm was used for the minimization process which performs 1000 steps of Steepest Descent with a RMS gradient tolerance of 3, followed by Conjugate Gradient minimization for faster convergence towards a local minimum [86]. Structure evaluations were carried out using DOPE, which is an atomic based statistical potential in MODELER package for model evaluation and structure prediction [87]. Structure verifications were carried out using VerifyProtein-Profiles-3D that allows evaluating the fitness of a protein sequence in its current 3D environment [49, 88, 89].

Virtual screening of metabolites that can bind to transcription factors was performed using the Discovery studio version 3.0, an integrated modeling and simulation solution for both small molecule and biotherapeutics-based research; and the version 3.0 used in this study has been released in December 2010 (Accelrys Inc, USA). The LibDock algorithm, an interface to the Discovery studio docking program was used for docking the metabolites into the binding pockets of the transcription factors. LibDock uses protein site features referred to as HotSpots. HotSpots consist of two types: polar and apolar. A polar Hotspot is preferred by a polar ligand atom (for example a hydrogen bond donor or acceptor) and an apolar HotSpot is preferred by an apolar atom (for example a carbon atom). The receptor HotSpot file is calculated prior to the docking procedure. The poses are pruned and a final optimization step is performed before the poses are scored [58]. To shortlist the strong binding metabolites, the HYDE scoring function in the LeadIT software suite was employed. LeadIT version 2.0.1 is an interactive graphical user interface, which embeds both docking and fragment-based design tools, FlexX and ReCore respectively, released in March 2011 (BioSolveIT GmbH, Germany). HYDE scoring can be applied to wide variety proteins since it is not calibrated on protein-ligand complexes [59].

### Availability of supporting data

The data sets supporting the results of this article are included within the article and its additional files.

## Additional files

Additional file 1:
**Chemical description of the 10 sugar molecules used in the study.** (XLSX 36 kb) Additional file 2:
**The number of genes per GO term found to be significantly different in expression between each of the 10 sugars and glucose.** (XLSX 15 kb) Additional file 3:
**The fraction of the total number of genes (that a pathway consists) that was found significantly up- and/or down- regulated in each sugar compared to glucose for 66 metabolic pathways.** (XLSX 46 kb) Additional file 4:
**Significant transcripts of Carbohydrate Active Enzymes (CAZy) by the presence of 10 different model oligosaccharides.** Regulation is indicated by arrows. (XLSX 19 kb) Additional file 5:
**List of significant genes expressing Carbohydrate Active enzymes (CAZy) and associated genes with same regulation profile.** (XLSX 15 kb) Additional file 6:
**Threading templates used for multi template modeling of the** ***A. oryzae*** **transcription factors viz., AoXlnR, AoAmyR and AoCreA along with their secondary structure alignments.** Structure evaluation scores for modeled transcription factors of the *A. oryzae* transcription factors viz., AoXlnR, AoAmyR and AoCreA. (XLSX 36 kb) Additional file 7:
**Additional details on the structures of XlnR, AmyR and CreA.** Supplementary Figures S1 to S7. (PDF 1780 kb) Additional file 8:
**Binding pockets in the structures of AoXlnR and AoAmyR with DoG score ≥ 0.8.** (XLSX 10 kb)

## Abbreviations

CAZy: carbohydrate active enzymes
CDH: cellobiose dehydrogenase
CE: carbohydrate esterase
GH: glycoside hydrolase
GT: glycosyl transferase
O-AHP: arabinoheptaose
O-BGHEXA: 1,3:1,4-β-Glucohexaose
O-CHE: cellohexaose
O-GM3: 6^1^-a-D-Galactosyl-mannotriose
O-GMH: 6^3^-a-D-Glucosyl-maltotriosyl-maltotriose
O-MHE: mannohexaose
O-X3G4R: xyloglucan (X_3_Glc_4_-Borohydride reduced)
O-XPE: xylopentaose
PL: polysaccharide lyase
PMO: polysaccharide monooxygenases
SOP: sophorose
TYR: turanose
