# Supplementary material for: Deciphering the signaling mechanisms of the plant cell wall degradation machinery in Aspergillus oryzae
Source: BMC Syst Biol. 2015 Nov 14;9:77. doi: 10.1186/s12918-015-0224-5 (PMC4647334; doi:10.1186/s12918-015-0224-5)
Supplement: Additional file 7: — Additional details on the structures of XlnR, AmyR and CreA. Supplementary Figures S1 to S7. (PDF 1780 kb) [file 12918_2015_224_MOESM7_ESM.pdf]

Structure of the Xylanolytic transcriptional activator XlnR:

Amino acid sequence alignment of the xylanolytic transcriptional activators from *A. niger* and *A. oryzae* showed high sequence similarity with 77% sequence identity (Supplementary Figure S1). The conserved functional domains of both AoXlnR and AnXlnRs are shown at primary structure level in Supplementary Figure S1, whereas AoXlnR is shown at tertiary structure level in Supplementary Figure S2A. The modeled structure of AoXlnR resembles a 'fisted' right hand [1] with thumb, fingers and palm subdomains (Supplementary Figure S2B-E). The finger subdomain is mainly formed by the putative DNA binding domain (in the area of folded little finger), a C-terminal coiled-coil (in the area of folded ring finger) and the inhibitory domain (covering the areas of folded middle and index fingers). The N-terminal coiled-coil region and a beta sheet loop (highlighted in red in Supplementary Figure S2E) that we believe to be involved in the structural rearrangement in the DNA binding process falls under the palm region. The thumb subdomain covers the part of the protein that connects the palm and the coiled regions of the fingers subdomain. The fisted structure of the AoXlnR resembles a 'grasping hand' to accommodate the promoter region of DNA during the binding process with major conformational changes in the coiled-coil region loops of the palm subdomain.

The consensus sequence of Cys-X_2-Cys-X_6-Cys-X_5-Cys-X_2-Cys-X_6-Cys that represents the putative DNA binding domain [2] can be found near the N-terminal region of AoXlnR. The presence of putative coiled-coil domain near the DNA-binding domain and a second coiled-coil region at the C-terminal end of *A. niger* XlnR (AnXlnR) has been predicted previously [3]. The N-terminal amphipathic α-helix formed by the hydrophobic residues is the main component of the N-terminal coiled-coil domain. In addition to two loss-of-function mutations, the presence of an activation domain has been suggested at the C-terminal region of AnXlnR. The coiled-coil domain in the C-terminal has been identified to be involved in the nuclear import of the protein and the downstream portion of the C-terminal coiled-coil domain has been shown to be involved in transcriptional regulation [4]. Contrasting observations by Hasper *et al* [4] in connection with the induction of xylanolytic genes and xylanase activities by XlnR mutants of *A. niger* led to the suggestion that the regulation mechanism of XlnR involves inter- or intramolecular interactions within the C-terminal domain. It has also been showed that D-xylose acts both an inducing and repressing carbon source for XlnR-dependent expression of xylanolytic genes in *A. niger* [5, 6]. Another study by Noguchi *et al* [7] showed that D-xylose-triggered reversible phosphorylation controls XlnR activity and further indicated that XlnR might change its preferential recognition sequence slightly depending on the inducer or metabolite. So, the intermolecular-intramolecular interaction domain plays an important role in controlling the XlnR regulatory relationships.

Structure of the Amylolytic transcriptional activator AmyR:

A. oryzae AmyR possess the functional domains homologous to *A. nidulans* AmyR (AnAmyR). AoAmyR showed 72% overall amino acid sequence identity with AnAmyR (Supplementary Figure S3). The four functional domains of AoAmyR are a cys-6 zinc cluster DNA binding domain at the N-terminus (residues 25-66), a trans-activation domain (residues 155-217), an inhibitory domain (239-380) and two inducer responsive domains (residues 416-493; residues 513-539). The zinc binuclear cluster DNA binding domain showed 100% sequence identity, indicating that AmyRs in *Aspergillus* bind to identical DNA sequence motifs [8]. A leucine zipper-like heptad repeat motif [9, 10], Leu-X6-Leu-X6-Leu-X6-Leu (residues 351-380), was found at the C-terminal end of the inhibitory domain. The transcriptional activation of genes involved in plant polysaccharide degradation is regulated by the trans-activation domain. The nuclear localization signal present in the N-terminal region and the co-ordinated action of both inducer responsive domains regulates the localization of AmyR in nucleus and cytoplasm [11].

A. oryzae AmyR (AoAmyR) is exclusively helical and contains twelve HEAT domains arranged into continuous orthogonal arches (Supplementary Figure S4A). The right-handed super-helix orthogonal arrangement of the arches colored according to the heat repeat domains is shown in Supplementary Figure S4B. The HEAT domains generally consists of repeats of two anti-parallel α-helices and two turns arranged about a common axis. HEAT repeats are linked by flexible inter-unit loops [12]. The HEAT repeat domains viz., HD1, HD2, HD3, HD5, HD6, HD7, HD9, HD11 and HD12 of the AoAmyR with two anti-parallel α-helices resembles the organization of anti-parallel α-helices seen in Kap-β2 [13] and protein phosphatase 2A [14]. The remaining HEAT repeat domains viz., HD4, HD8 and HD10 with more than two α-helices resembles the armadillo repeats in Kap-α [15] and β-catenin where its 12 repeats form a superhelix of alpha helices with three helices per unit [16]. The α-helices (α1, α2, α3....) and the inter-unit loops (L1, L2, L3....) present in each HEAT domain of AoAmyR are shown in Supplementary Figure S5. The long loop L5 connects the helices of the HEAT domain 4 and spans both orthogonal arches of the protein structure (Supplementary Figure S4A). The HEAT repeats with equivalent helices are parallel, with α2 helices towards the concave surface of AoAmyR structure and α1 helices lining the convex side or form the spine of the orthogonal arches. The presence of proline residues results to a bend in the middle of the helices in some HEAT domains while part of the helices are replaced by small loops. The presence of the bend/disordered helices and loops can also be seen in the proteins with HEAT domains and armadillo repeats [12-15]. The helical repeats of AoAmyR provide several spatially distinct binding sites for interactions with various ligands.

Structure of the Carbon catabolite repressor CreA:

An interesting feature of the modeled *A. oryzae* CreA (AoCreA) is that it resembles the structure of the multidomain complement protein properdin. Properdin is known to exist in different arrangements of dimer and trimer structures. Properdins are highly flexible, elongated and narrow proteins with thrombospondin repeat domains [17]. Analysis of AoCreA at amino acid sequence level indicated the presence of the classic C_2H_2 Zinc finger domains throughout the length, which further complicates our understanding on the functional domains of CreA. As shown in Supplementary Figure S6, sequence analysis using HMMER [18] and IMPALA [19] indicated that the N-terminal residues 72-128 forms the C_2H_2 Zinc finger domains that are relatively small protein motifs, which contain multiple finger-like protrusions that make tandem contacts with their target molecule. Sequence analysis using RPS-BLAST [20] showed that the region spanning the residues 43-311, as COG5048 Zinc finger domain. The length of the AoCreA is 429 amino acid residues out of which the region spanning 316 residues is predicted as Zinc finger domain. The presence of a large region with zinc finger gives the flexibility to the CreA protein structure and indicates the complexity of its regulation mechanism, since zinc finger motifs are well known to fold in many occasions to mediate independent intermolecular interactions between nucleic acids (DNA and RNA), proteins and lipids [21-23]. CreA has been shown to be involved in the regulation of proline, ethanol, xylan and arabinan utilization [24] but it also regulates the expression of the AmyR gene that encodes the trasncriptional acticator for the amylase genes [8]. So the analysis of the sequence and structural features of AoCreA will contribute to our understanding on the often underestimated versatility of the CreA transcriptional regulation. Due to the poor knowledge of the functional domains in AoCreA and limitations to simulate the dimer or trimer CreA structures, in the current study we focused on analyzing the AoXlnR and AoAmyR-metabolite interactions.

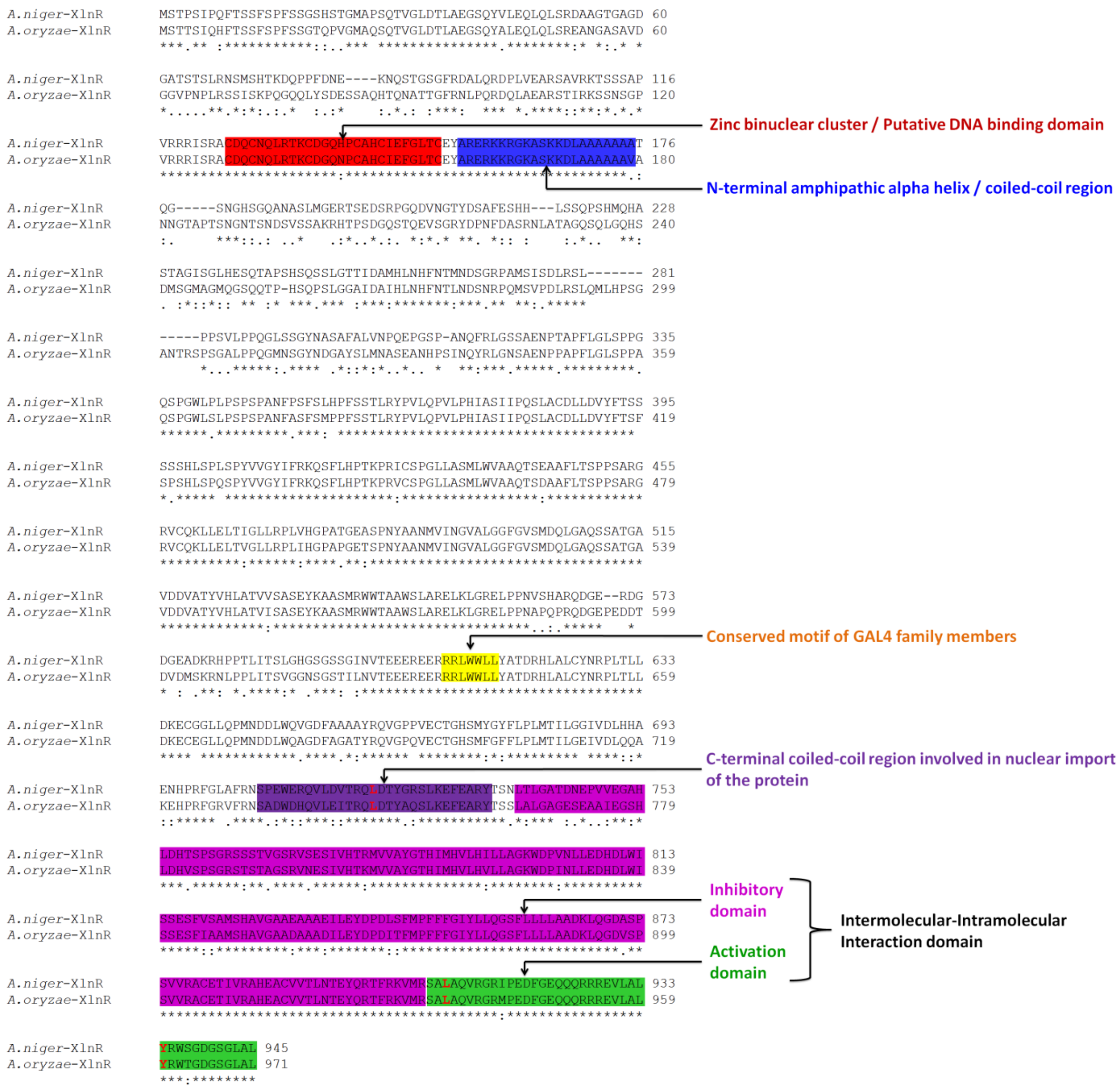

Supplementary Figure S1: The consensus domains of the xylanolytic transcriptional activators from *A. niger* and *A. oryzae* depicted on the primary sequence alignment.

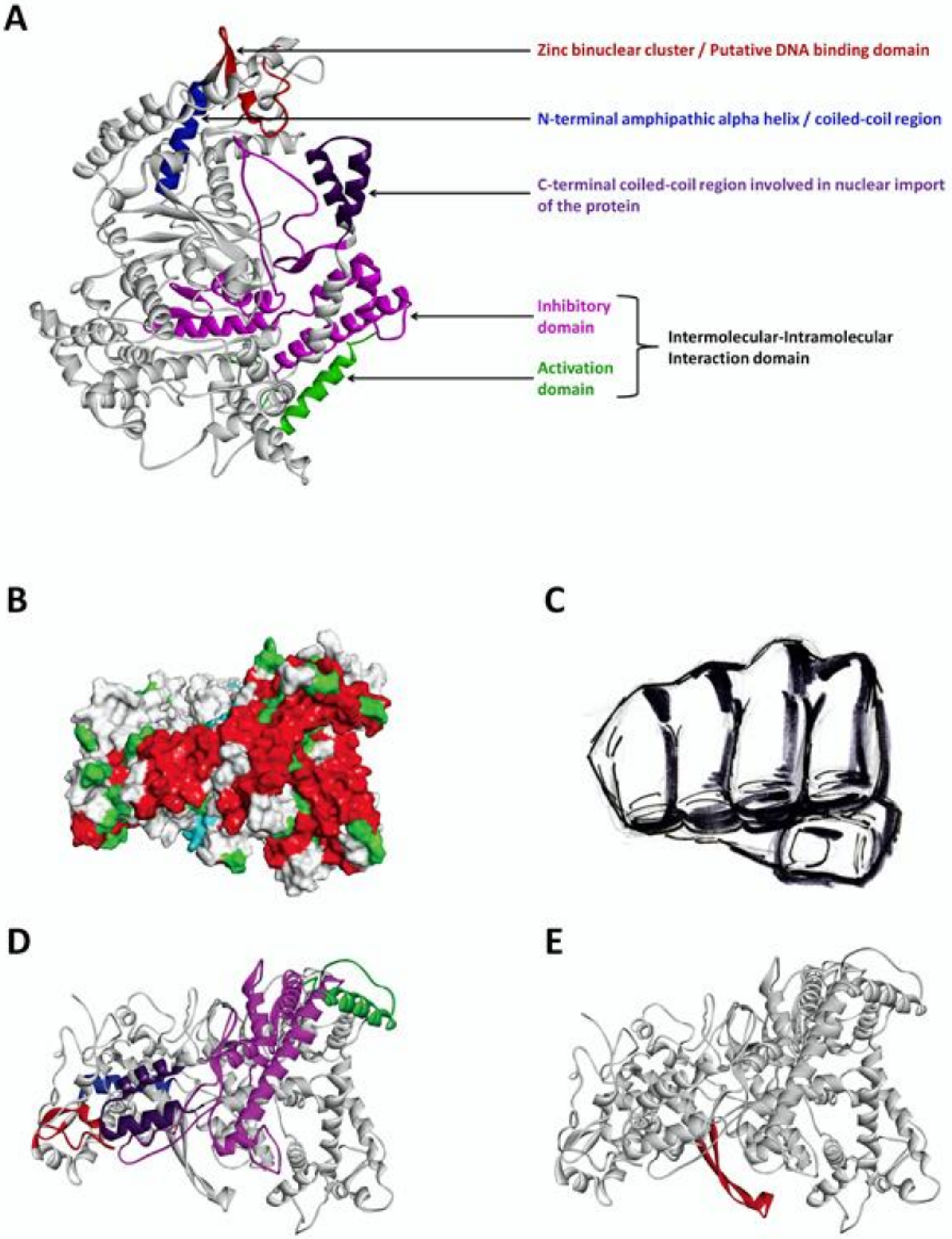

Supplementary Figure S2: The modeled 3D structure of the *A. oryzae* xylanolytic transcriptional activator (AoXlnR). The figure shows the structural resemblance of the *A. oryzae* xylanolytic transcriptional activator with the right hand fist. (A) The conserved functional domains of AoXlnR were highlighted on the ribbon structure in different colors. (B) Solvent surface rendered structure of the modeled AoXlnR colored according to the secondary structure. Red represents alpha helix regions; Cyan represents beta strand regions; Green represents coil regions. (C) A sketch of the right hand fist. (D) Ribbon representation of the AoXlnR structure. The activation domain and inhibitory domains are highlighted in magenta and green colors, respectively. The putative DNA binding domain is shown in red. The N-terminal coiled-coil region is highlighted in violet blue, whereas the C-terminal coiled-coil region is colored in purple. (E) The ribbon structure of AoXlnR with the beta sheet loop shown in red.

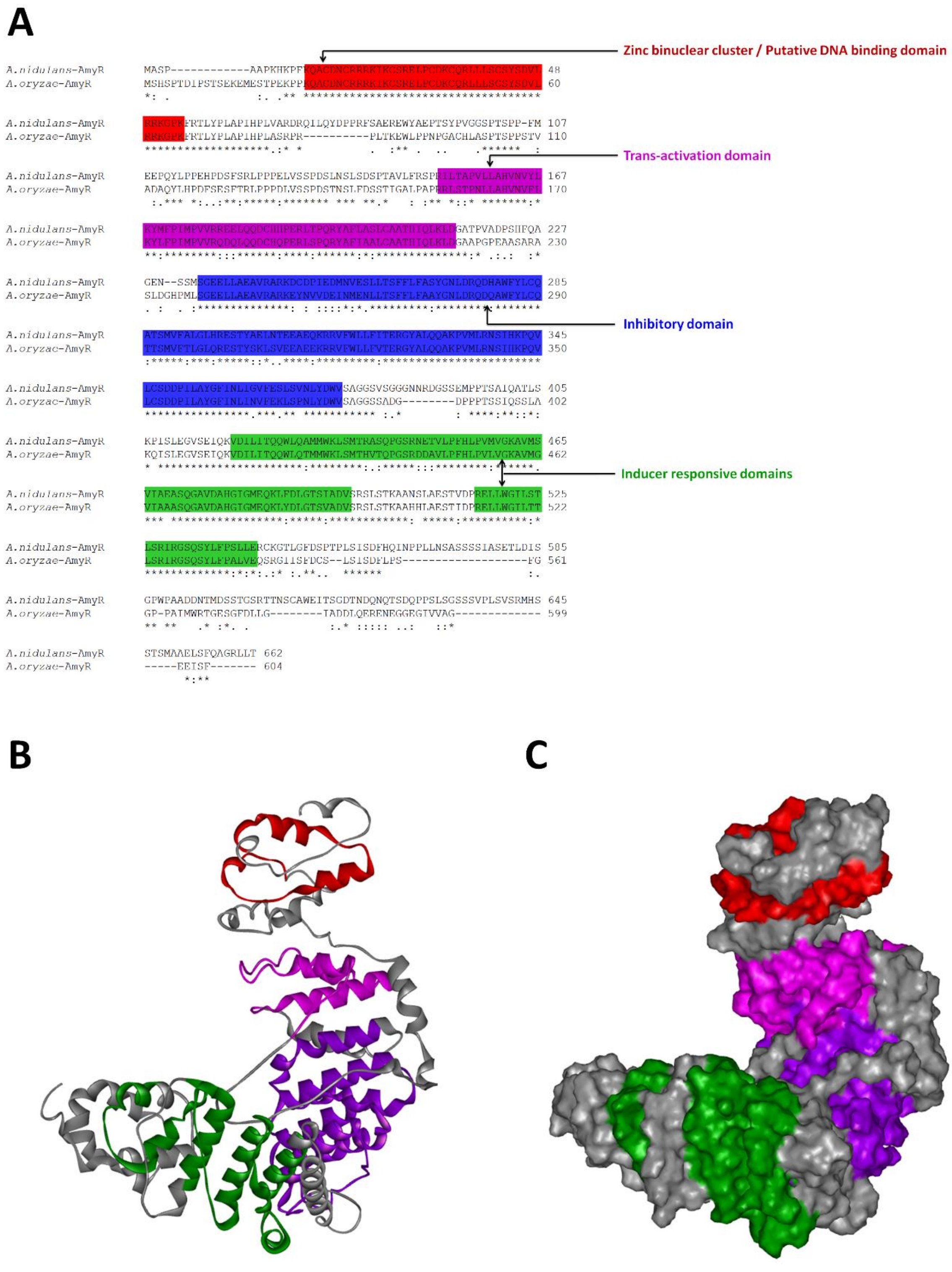

Supplementary Figure S3: Functional domains of *A. oryzae* amylolytic transcriptional activator (AoAmyR). (A) The consensus domains of AmyR proteins from *A. niger* and *A. oryzae* depicted on the primary sequence alignment. Modeled 3D structure of AoAmyR shown in ribbon representation (B) and solvent surface representation (C) with the functional domains highlighted in different colors. The trans-activation domain and inhibitory domains are highlighted in magenta and violet blue colors respectively. The putative DNA binding domain comprising is shown in red. The inducer responsive domain is colored in green.

A

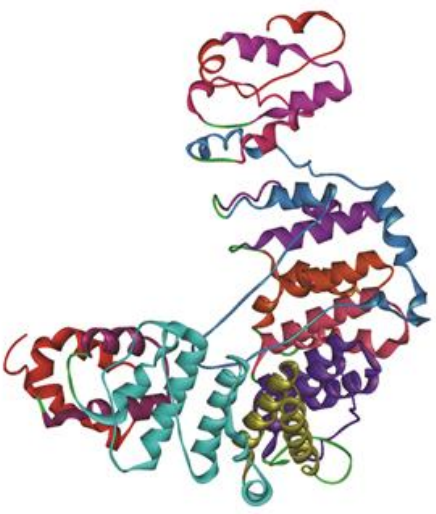

B

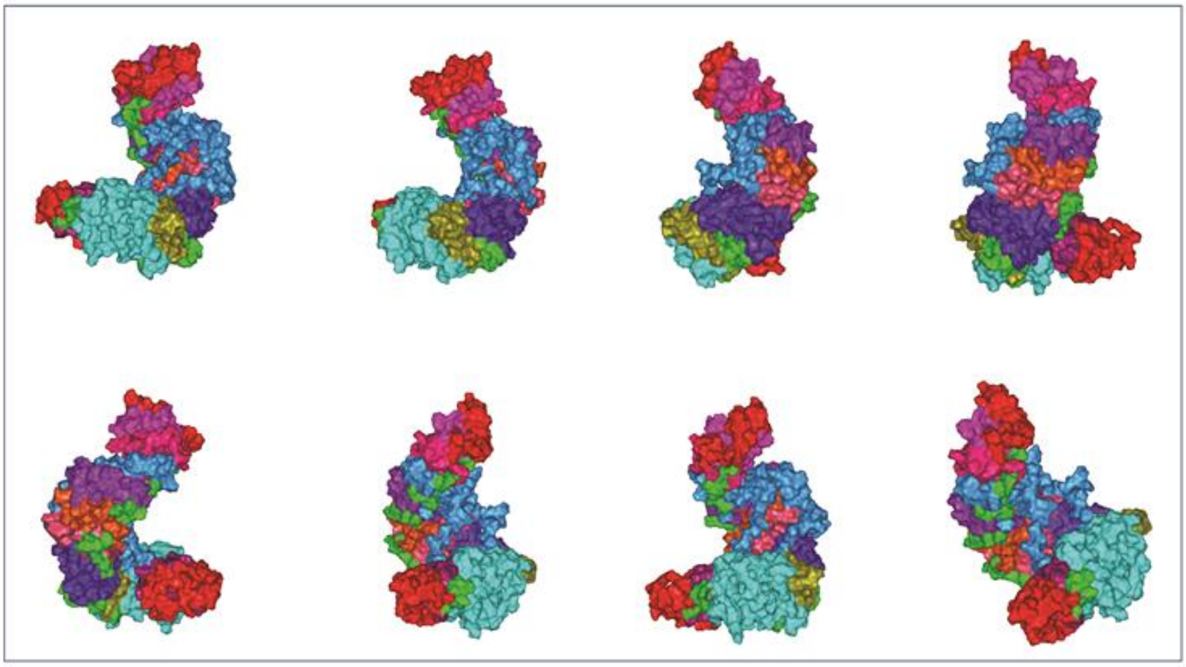

Supplementary Figure S4: Structure of *A. oryzae* amylolytic transcriptional activator (AoAmyR). (A) The ribbon structure representation of AoXlnR with twelve HEAT domains highlighted in different colors. (B) Different rotational screenshots of AoXlnR representing the right-handed superhelix orthogonal arrangement of the arches.

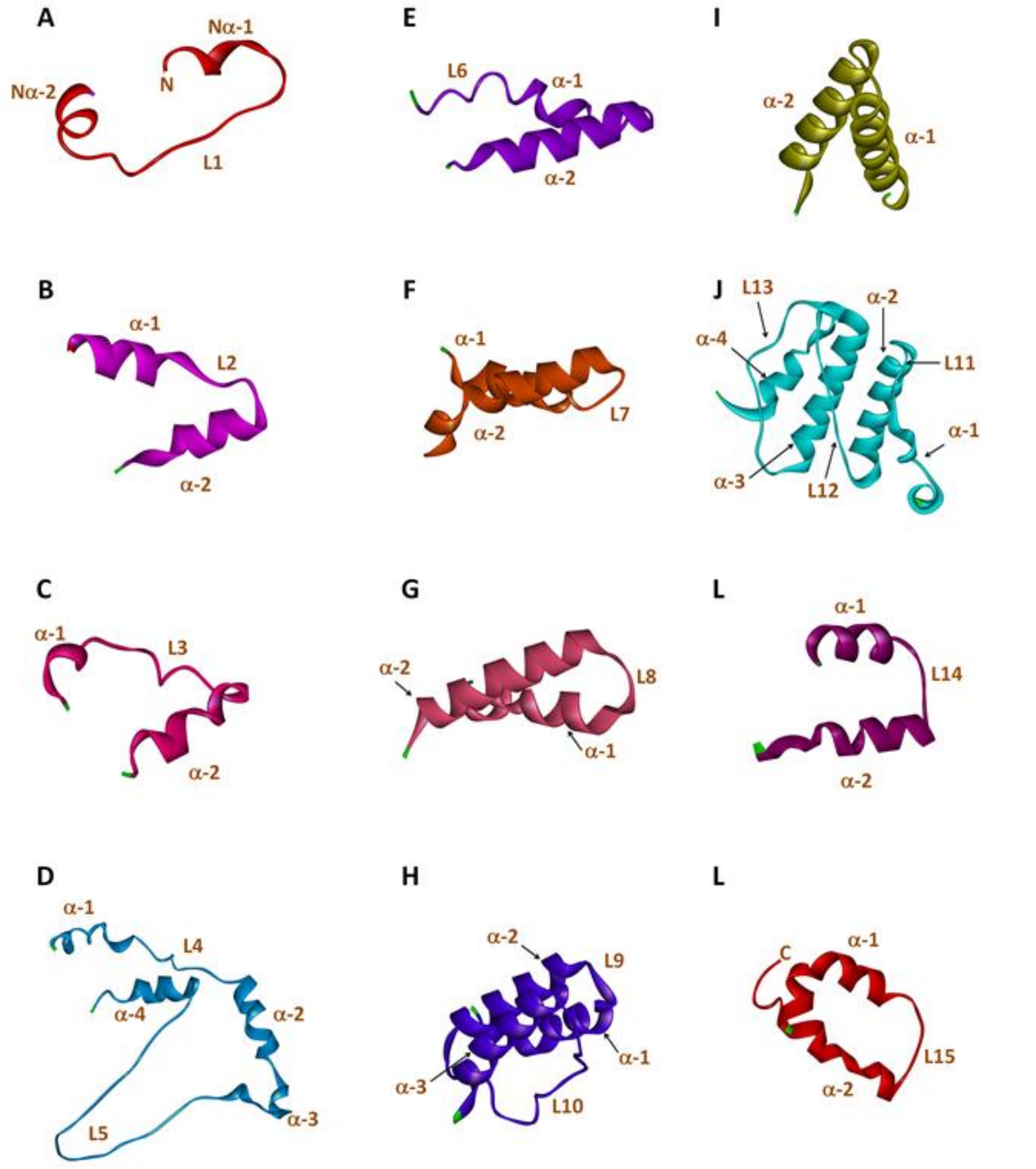

Supplementary Figure S5: The α-helices (α1, α2, α3....) and the inter-unit loops (L1, L2, L3....) present in each HEAT repeat domain of AoAmyR. The HEAT repeat domains viz., HD1, HD2, HD3, HD5, HD6, HD7, HD9, HD11 and HD12 possess of two anti-parallel α-helices. The HEAT repeat domains viz., HD4, HD8 and HD10 contain more than two α-helices. (A) HEAT repeat domain 1. HD1 located at the N-terminal end contains two alpha helices viz., Nα1 and Nα2 are connected by loop L1. (B) HEAT repeat domain 2. HD2 contains two alpha helices connected by loop L2. (C) HEAT repeat domain 3. HD3 contains two alpha helices connected by loop L3. (D) HEAT repeat domain 4. HD4 comprises of four aplha helices. α1 and α2 are connected by loop L4. A short stretch of sequence connects α2 and α3 helices. A hairpin loop L5 connects the helices α3 and α4. (E) HEAT repeat domain 5. HD4 and HD5 are connected through loop L6, whereas the two α-helices of HD5 are connected by a short stretch of protein sequence. (F) HEAT repeat domain 6. HD6 contains two alpha helices connected by loop L7. (G) HEAT repeat domain 7. HD7 contains two alpha helices connected by loop L8. (H) HEAT repeat domain 8. HD8 contains three alpha helices viz., α1, α2 and α3. Loop L9 connects α1 and α2 helices of HD8, whereas loop L10 connects α2 and α3 helices (I) HEAT repeat domain 9. The two α-helices of HD9 are connected by a short stretch of protein sequence. (J) HEAT repeat domain 10. HD10 contains four alpha helices viz., α1, α2, α3 and α4. Helices α1 and α2 are connected by loop L11. Helices α2, α3 and α4 are interconnected by loops loops L12 and L13. (K) HEAT repeat domain 11. HD11 contains two alpha helices connected by loop L14. (L) HEAT repeat domain 12. HD12 located at the C-terminal end contains two alpha helices connected by loop L15.

HMMER alignment against: ZnF_C2H2 domain 1

```
                   *->ykCpigCgksfssk.aLkrHmrvH<-*
                      ykCp+ C++ f++ ++ +rH+r+H
      AoCreA    76    YKCPL-CDRAFHRLeHQTRHIRTH    98
```

HMMER alignment against: ZnF_C2H2 domain 2

```
                   *->ykCpi.gCgksfssk.aLkrHmrvH<-*
                      + C+++gC k+fs+ ++L+rH r+H
      AoCreA   104    HACQFpGCTKRFSRSdELTRHSRIH    128
```

IMPALA-Alignment against: ZnF_C2H2 domain

```
AoCreA: 72  LPRPYKCPLCDRAFHRLEHQTRHIRT---HTGEKPHACQFPGCT---KRFSRSDELTRHS 125
            +        C   F   +   +HI T   H  +K   C+  GC+   K F     L  H
        4   IETNCHWVDCKLEFPTQDDLVKHINTDHIHASKKAFVCRRVGCSRDEKPFKAQYMLVVHM 63

AoCreA: 126 RIHNN 130
            R H
        64  RRHTG 68
```

RPS-BLAST-Alignment against: COG5048 Zn-finger

```
AoCreA: 43  SNSTMASSVSLLPPLMKGARPATEEARQDLPRPYKCPLCDRAFHRLEHQTRHIRTHTGEK 102
            +  T + S S    ++     +T ++  + PRP  CP C  +F RLEH TRHIR+HTGEK
        1   ATLTSSQSSSSNNSVLSSTPKSTLKSLSNAPRPDSCPNCTDSFSRLEHLTRHIRSHTGEK 60

AoCreA: 103 PHACQFPGCTKRFSRSDELTRHSRIHNNPNSRRSNKAHLAAAAAAAAAGQENAMVNVTNA 162
            P  C + GC K FSR  EL+RH R H+N  S  ++K+   + + A+++   ++  N  +
        61  PSQCSYSGCDKSFSRPLELSRHLRTHHNNPSDLNSKSLPLSNSKASSSSLSSSSSNSNDN 120

AoCreA: 163 GSLMPPPTKPMTRSAPVSQVGSPDVSPPHSFSNYAGHMRSNLGPYARNTERASSGMDINL 222
              L      P +R   +     PD+    +  N      ++       +      +  N
        121 NLLSSHSLPPSSRDPQL-----PDLLSISNLRNNPLPGNNSSSVNTPQSNSLHPPLPANS 175

AoCreA: 223 LATAASQVERDEQHFGFHAGPRNHHLFASRHHTGRGLPSLSAYAISHSMSRSHSHEDEDG 282
            L+   S                +    +    +     S S   + +S S      +
        176 LSKDPSSNLSLLISSNVSTSIPSSSENSPLSSSYSIPSSSSDQNLENSSSSLPLTTNSQL 235

AoCreA: 283 YTHRVKRSRPNSPNSTAPSSPTFSHDSLSPTPDHTPLATPAHSPRLRPLGSSELHLPSIR 342
                +    P+S +S+  SS        S     +  ++P  S      G S
        236 SPKSLLSQSPSSLSSSDSSSSASESPRSSLPTASSQSSSPNESDSSSEKGFSLPIKSKQC 295

AoCreA: 343 HLSLHHTPALAPMEPQ 358
            ++S   +  L
        296 NISFSRSSPLTRHLRS 311
```

Supplementary Figure S6: Zing finger domains that are spread along the protein sequence of *A. oryzae* Carbon catabolite repressor (AoCreA).

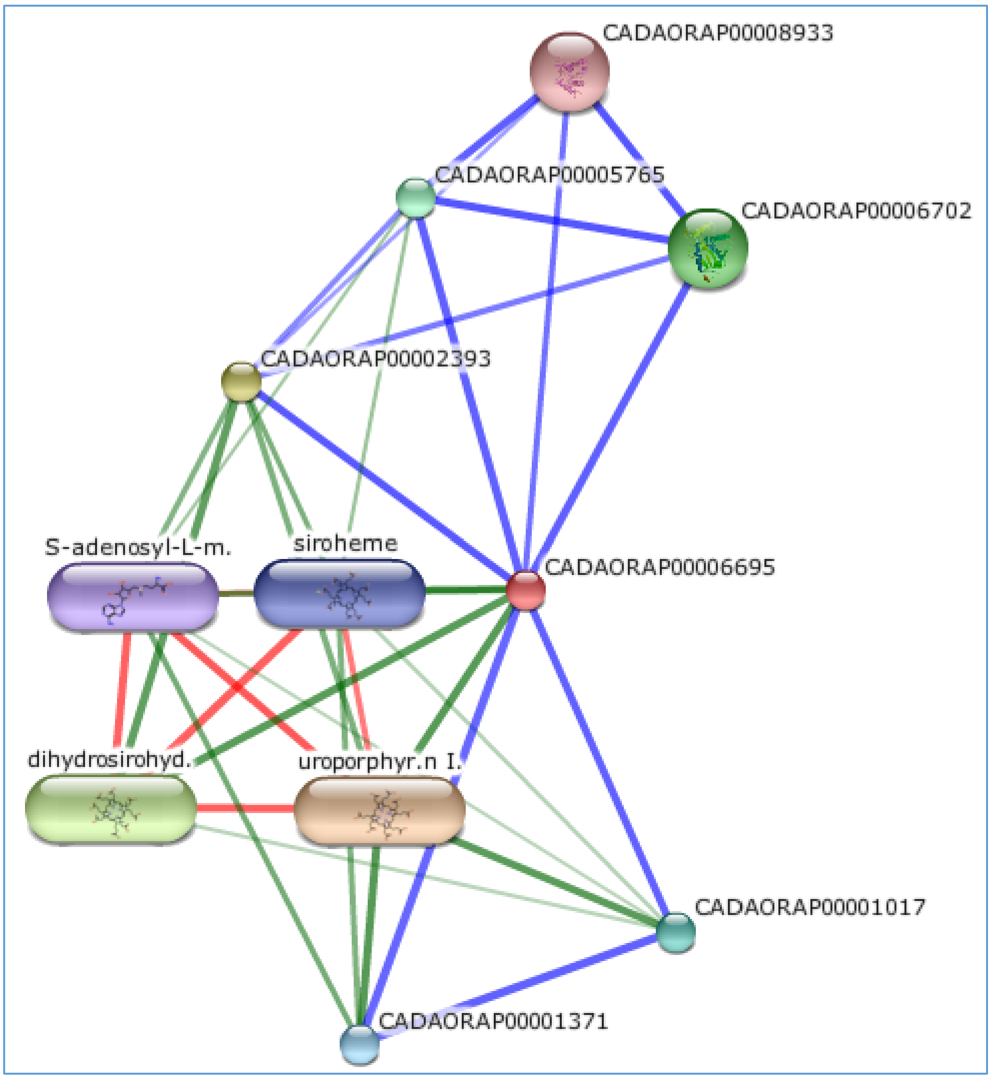

Predicted Functional Partners:

		Score
uroporphyr.n I.	uroporphyrinogen III (836.8 g/mol)	0.987
CADAORAP00002393	Predicted protein (235 aa)	0.978
dihydrosirohyd.	dihydrosirohydrochlorin (864.8 g/mol)	0.973
CADAORAP00006702	Phosphoadenosine phosphosulfate reductase (308 aa)	0.941
CADAORAP00005765	Sulfite reductase (1527 aa)	0.933
CADAORAP00001017	Uroporphyrinogen III synthase UROS/HEM4 (309 aa)	0.932
CADAORAP00001371	Uroporphyrinogen decarboxylase (378 aa)	0.899
siroheme	The heme prosthetic group of the hemoprotein of e coli nadph-sulfite reductase; reduces sulfite [...] (916.7 g/mol)	0.897
S-adenosyl-L-m.	S-adenosyl-L-methionine; S-Adenosyl methionine (SAM, SAMe, SAM-e) is a common co-substrate invo [...] (399.4 g/mol)	0.871
CADAORAP00008933	Adenylyl-sulfate kinase (EC 2.7.1.25); Catalyzes the synthesis of activated sulfate (By similar [...] (211 aa)	0.838

Supplementary Figure S7:-protein interaction network of strong binding metabolites and the enzymes catalyzing the associated reactions. The score (0 to 1) depicts the evidence available in the literature for the respective functional partners.

References

1. Murakami KS, Davydova EK, Rothman-Denes LB: **X-ray crystal structure of the polymerase domain of the bacteriophage N4 virion RNA polymerase**. *Proceedings of the National Academy of Sciences of the United States of America* 2008, **105**(13):5046-5051.
2. Marmorstein R, Carey M, Ptashne M, Harrison SC: **DNA recognition by GAL4: structure of a protein-DNA complex**. *Nature* 1992, **356**(6368):408-414.
3. Lupas A, Van Dyke M, Stock J: **Predicting coiled coils from protein sequences**. *Science* 1991, **252**(5009):1162-1164.
4. Hasper AA, Trindade LM, van der Veen D, van Ooyen AJ, de Graaff LH: **Functional analysis of the transcriptional activator XlnR from Aspergillus niger**. *Microbiology* 2004, **150**(Pt 5):1367-1375.
5. de Vries RP, van den Broeck HC, Dekkers E, Manzanares P, de Graaff LH, Visser J: **Differential expression of three alpha-galactosidase genes and a single beta-galactosidase gene from Aspergillus niger**. *Applied and environmental microbiology* 1999, **65**(6):2453-2460.
6. de Vries RP, Visser J, de Graaff LH: **CreA modulates the XlnR-induced expression on xylose of Aspergillus niger genes involved in xylan degradation**. *Research in microbiology* 1999, **150**(4):281-285.
7. Noguchi Y, Tanaka H, Kanamaru K, Kato M, Kobayashi T: **Xylose triggers reversible phosphorylation of XlnR, the fungal transcriptional activator of xylanolytic and cellulolytic genes in Aspergillus oryzae**. *Bioscience, biotechnology, and biochemistry* 2011, **75**(5):953-959.
8. Tani S, Katsuyama Y, Hayashi T, Suzuki H, Kato M, Gomi K, Kobayashi T, Tsukagoshi N: **Characterization of the amyR gene encoding a transcriptional activator for the amylase genes in Aspergillus nidulans**. *Current genetics* 2001, **39**(1):10-15.
9. Landschulz WH, Johnson PF, McKnight SL: **The leucine zipper: a hypothetical structure common to a new class of DNA binding proteins**. *Science* 1988, **240**(4860):1759-1764.
10. Gomi F, Imaizumi K, Yoneda T, Taniguchi M, Mori Y, Miyoshi K, Hitomi J, Fujikado T, Tano Y, Tohyama M: **Molecular cloning of a novel membrane glycoprotein, pal, specifically expressed in photoreceptor cells of the retina and containing leucine-rich repeat**. *The Journal of neuroscience : the official journal of the Society for Neuroscience* 2000, **20**(9):3206-3213.
11. Makita T, Katsuyama Y, Tani S, Suzuki H, Kato N, Todd RB, Hynes MJ, Tsukagoshi N, Kato M, Kobayashi T: **Inducer-dependent nuclear localization of a Zn(II)(2)Cys(6) transcriptional activator, AmyR, in Aspergillus nidulans**. *Bioscience, biotechnology, and biochemistry* 2009, **73**(2):391-399.
12. Vetter IR, Arndt A, Kutay U, Gorlich D, Wittinghofer A: **Structural view of the Ran-Importin beta interaction at 2.3 A resolution**. *Cell* 1999, **97**(5):635-646.
13. Chook YM, Blobel G: **Structure of the nuclear transport complex karyopherin-beta2-Ran x GppNHp**. *Nature* 1999, **399**(6733):230-237.
14. Groves MR, Hanlon N, Turowski P, Hemmings BA, Barford D: **The structure of the protein phosphatase 2A PR65/A subunit reveals the conformation of its 15 tandemly repeated HEAT motifs**. *Cell* 1999, **96**(1):99-110.
15. Conti E, Uy M, Leighton L, Blobel G, Kuriyan J: **Crystallographic analysis of the recognition of a nuclear localization signal by the nuclear import factor karyopherin alpha**. *Cell* 1998, **94**(2):193-204.
16. Huber AH, Nelson WJ, Weis WI: **Three-dimensional structure of the armadillo repeat region of beta-catenin**. *Cell* 1997, **90**(5):871-882.
17. Sun Z, Reid KB, Perkins SJ: **The dimeric and trimeric solution structures of the multidomain complement protein properdin by X-ray scattering, analytical ultracentrifugation and constrained modelling**. *Journal of molecular biology* 2004, **343**(5):1327-1343.

18. Schultz J, Milpetz F, Bork P, Ponting CP: **SMART, a simple modular architecture research tool: Identification of signaling domains**. *P Natl Acad Sci USA* 1998, **95**(11):5857-5864.
19. Schaffer AA, Wolf YI, Ponting CP, Koonin EV, Aravind L, Altschul SF: **IMPALA: matching a protein sequence against a collection of PSI-BLAST-constructed position-specific score matrices**. *Bioinformatics* 1999, **15**(12):1000-1011.
20. Ooi HS, Kwo CY, Wildpaner M, Sirota FL, Eisenhaber B, Maurer-Stroh S, Wong WC, Schleiffer A, Eisenhaber F, Schneider G: **ANNIE: integrated de novo protein sequence annotation**. *Nucleic acids research* 2009, **37**:W435-W440.
21. Matthews JM, Sunde M: **Zinc fingers - Folds for many occasions**. *Iubmb Life* 2002, **54**(6):351-355.
22. Hall TMT: **Multiple modes of RNA recognition by zinc finger proteins**. *Curr Opin Struc Biol* 2005, **15**(3):367-373.
23. Brown RS: **Zinc finger proteins: Getting a grip on RNA**. *Curr Opin Struc Biol* 2005, **15**(1):94-98.
24. Ruijter GJG, Visser J: **Carbon repression in Aspergilli**. *Fems Microbiol Lett* 1997, **151**(2):103-114.
